# ORFik: a comprehensive R toolkit for the analysis of translation

**DOI:** 10.1186/s12859-021-04254-w

**Published:** 2021-06-19

**Authors:** Håkon Tjeldnes, Kornel Labun, Yamila Torres Cleuren, Katarzyna Chyżyńska, Michał Świrski, Eivind Valen

**Affiliations:** 1 Computational Biology Unit, Department of Informatics, University of Bergen, Bergen, Norway; 2 Sars International Centre for Marine Molecular Biology, University of Bergen, Bergen, Norway; 3 Institute of Genetics and Biotechnology, Faculty of Biology, University of Warsaw, Warsaw, Poland

**Keywords:** Analysis workflow, Translation, Translation initiation, 5′ UTRs, Open reading frames, uORFs, Ribo-seq, CAGE, RNA-seq, TCP-seq

## Abstract

**Background:**

With the rapid growth in the use of high-throughput methods for characterizing translation and the continued expansion of multi-omics, there is a need for back-end functions and streamlined tools for processing, analyzing, and characterizing data produced by these assays.

**Results:**

Here, we introduce ORFik, a user-friendly R/Bioconductor API and toolbox for studying translation and its regulation. It extends GenomicRanges from the genome to the transcriptome and implements a framework that integrates data from several sources. ORFik streamlines the steps to process, analyze, and visualize the different steps of translation with a particular focus on initiation and elongation. It accepts high-throughput sequencing data from ribosome profiling to quantify ribosome elongation or RCP-seq/TCP-seq to also quantify ribosome scanning. In addition, ORFik can use CAGE data to accurately determine 5′UTRs and RNA-seq for determining translation relative to RNA abundance. ORFik supports and calculates over 30 different translation-related features and metrics from the literature and can annotate translated regions such as proteins or upstream open reading frames (uORFs). As a use-case, we demonstrate using ORFik to rapidly annotate the dynamics of 5′ UTRs across different tissues, detect their uORFs, and characterize their scanning and translation in the downstream protein-coding regions.

**Conclusion:**

In summary, ORFik introduces hundreds of tested, documented and optimized methods. ORFik is designed to be easily customizable, enabling users to create complete workflows from raw data to publication-ready figures for several types of sequencing data. Finally, by improving speed and scope of many core Bioconductor functions, ORFik offers enhancement benefiting the entire Bioconductor environment.

**Availability:**

http://bioconductor.org/packages/ORFik.

**Supplementary Information:**

The online version contains supplementary material available at 10.1186/s12859-021-04254-w.

## Background

Messenger RNAs (mRNAs) can be divided into three regions: the transcript leader sequence also known as the 5′ untranslated region (5′ UTR), the coding sequence (CDS), and the trailer sequence or 3′ untranslated region (3′ UTR). Eukaryotic translation is normally initiated by the binding of the 40S ribosomal small subunit (SSU) and associated initiation factors, adjacent to the mRNA 5′ cap. The SSU then proceeds to scan downstream, until it reaches a favorable initiation context at the start of an open reading frame (ORF). Here, it recruits the 60S large ribosomal unit, which together with the SSU forms the 80S elongating complex which starts translating the protein. Inside the 80S ribosome there are three binding sites that accomodate tRNA base-pairing to mRNA: the aminoacyl-site (A), the peptidyl-site (P), and the exit-site (E). Incoming aminoacyl-tRNAs enter the ribosome at the A site, binding to the mRNA codon. The peptidyl-tRNA carrying the growing polypeptide chain is held in the P site, while the E site holds deacylated tRNAs just before they exit the ribosome (Additional file 1: Fig. S1). The 80S proceeds to translate the ORF, processing it codon-by-codon by translocating 3 nucleotides (nts) for each step, until it reaches a terminating codon and the protein synthesis is complete [1].

While eukaryotic transcripts typically encode only a single protein, evidence from high-throughput methods has revealed that many 5′ UTRs contain short upstream ORFs (uORFs) that can be translated [2]. While the functional importance of uORFs is still debated, several uORFs have been found to regulate gene expression [2, 3]. This primarily occurs by hindering ribosomes from reaching the protein-coding ORF leading to translational inhibition. This demonstrates that at least a subset of uORFs is functionally important.

While translation was previously studied on a gene-by-gene basis, the introduction of ribosome profiling (ribo-seq) and later, translation complex profiling (TCP-seq) and ribosome complex profiling (RCP-seq) has made it possible to obtain a snapshot of translating and scanning ribosomes across the whole transcriptome [4–6]. Together with information on RNA levels and isoforms, protein-coding ORFs and uORFs can be identified and their translational levels can be quantified. Getting functional insight from sequencing data requires robust computational analysis. Ribo-seq, being a mature assay, has a number of software packages and web services designed specifically to handle it. TCP-seq [5, 7, 8] and RCP-seq [6], on the other hand, are much less supported. These methods need tools that can consider both scanning SSU and 80S elongating ribosome dynamics as well as the relationship between these.

Another complicating factor is that many genes have alternative transcription start sites (TSSs) [9]. The study of translation initiation requires accurate 5′ UTRs annotation. It is otherwise challenging to determine which uORF candidates should be included in the analysis. The choice of TSS dictates which uORFs are present in the 5′ UTR. In certain cases, uORFs are only present in specific tissues with the correct variant of the 5′ UTR [10].

To address these challenges and provide a comprehensive tool for studying translation in custom regions, we developed ORFik, a Bioconductor software package that streamlines the analysis of translation. It supports accurate 5′ UTR annotation through RNA-seq and cap analysis of gene expression (CAGE), detection and classification of translated uORFs, characterization of sequence features, and the calculation of over 30 features and metrics used in the analysis of translation (Additional file 1: Table S1).

## Implementation

ORFik is implemented as an open-source software package in the R programming language, with parts in C++ to efficiently process large datasets. While tools for analyzing translation exist, none of them support comprehensive analysis of ribo-seq, TCP-seq and RCP-seq in combination with CAGE. Furthermore, many of these are either online tools [11], or are limited to studying only specific steps or aspects of translation [12]. A full comparison between functionality of related tools can be found in Additional file 1: Table S2 [13–23] and benchmarks comparing ORFik to related tools can be found in Additional file 1: Table S3-Table S5 and Additional file 1: Figs. S2–S4.

**Fig. 1:**
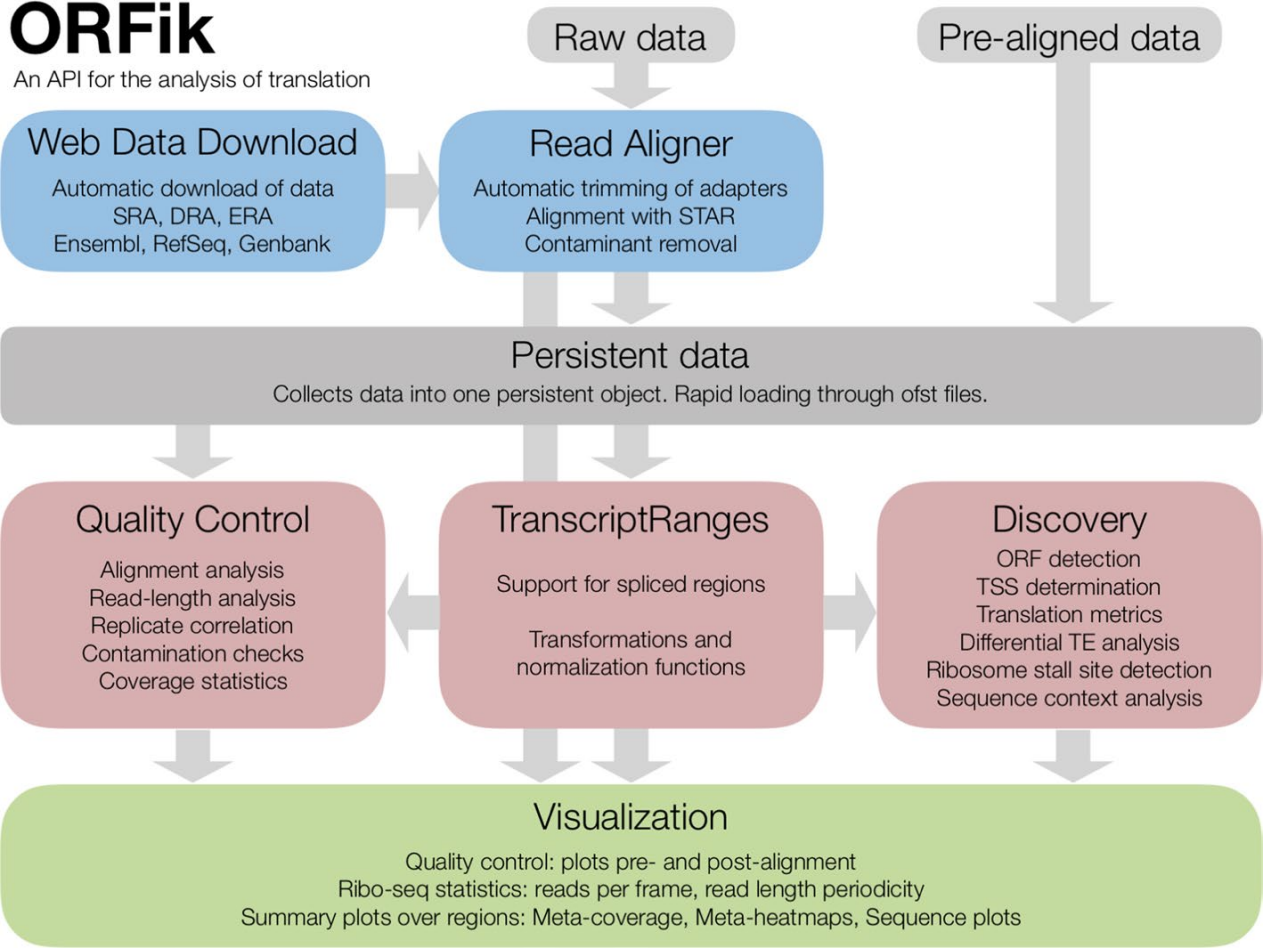
ORFik API overview. ORFik is a versatile API for the analysis of translation. It consists of 7 main components with gray arrows representing data flow through the components. Data is first loaded either directly from raw or pre-aligned form or through the Web Data Download API (left blue box). The latter allows for automatic downloading of data which increases replicability by allowing scripts that include the whole process from data retrieval to analysis. If not previously aligned, the Read Aligner API (right blue box) can trim adapters from fastq files, remove contaminants and align the reads, For this, ORFik uses fastp and STAR. Persistent Data (grey box) is then ensured through storage in a ORFik.experiment object which represents an experiment. This contains file paths to NGS data and genome annotation used. From this data can be retrieved to perform Quality Control (left red box). ORFik implements extensive quality control at several processing steps: contamination QC, alignment QC, annotation QC and analysis QC. For analyses, data is typically processed through the TranscriptRanges API (middle red box). This extends GenomicRanges to facilitate work with transcripts, supporting genomically disjoint regions like ORFs spanning several exons (described in Additional file 1: Table S6). At a more high level the Discovery API (right red box) provides tools that take advantage of TranscriptRanges to accomplish common tasks, among others: ORF finding, P-shifting and initiation context analysis over ORFs. Finally, results from the QC and analysis can be displayed through the Visualization API (green box). This supports a range of figures which can be normalized and summarized in a multitude of ways (described in Additional file 1: Table S1)

ORFik is highly optimized and fast. To achieve this, we have reimplemented several functions in the Bioconductor core package GenomicFeatures that are currently inefficient for larger datasets, like converting from transcript coordinates to genomic coordinates and vice versa. In addition, to aid with the ever-increasing size of datasets, we have focused on allowing faster computation of large bam files with our format “.ofst” based on the Facebook compression algorithm zstd [24, 25]. “.ofst” is a serialized format (see the section on Optimized File Format), with optional collapsing of duplicated reads, enabling near-instantaneous data loading (Fig. 1; Additional file 1: Table S5).

### Overview

**Fig. 2:**
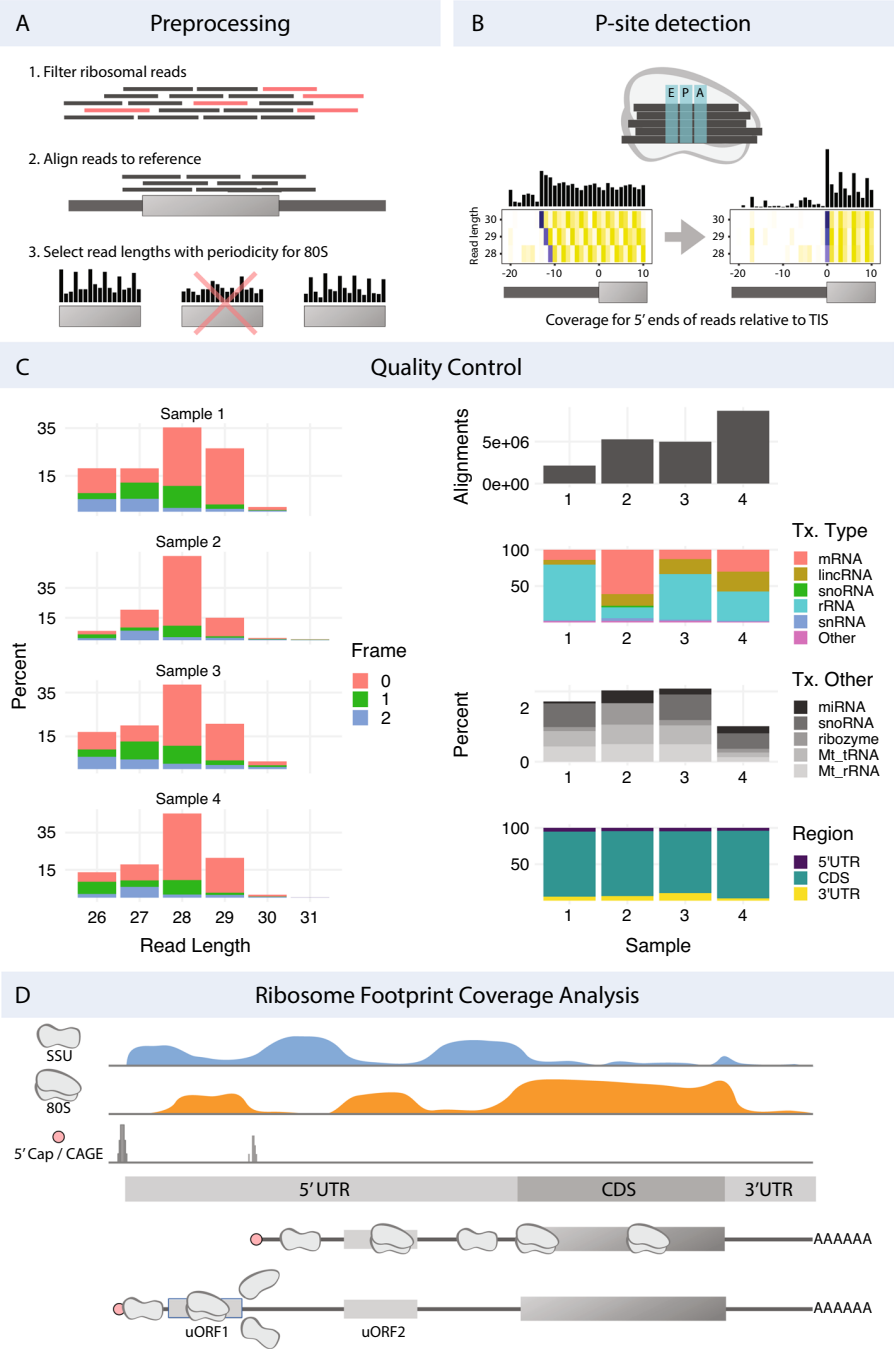
ORFik functionality. **a** ORFik supports a number of preprocessing steps including (1) removal of rRNA contamination) and other ncRNA), (2) Alignment to genome or transcriptome, (3) selecting 80S read lengths that display 3 nt periodicity characteristic of ribosome translocation over translated regions (see Additional file 1: Fig. S1). **b** To identify the P-sites for reads (top illustration) from an 80S library ORFik performs change point detection on the 5′ ends of reads across a window over translation initiation sites. This determines the location of reads from initiating ribosomes and the distance from their 5′ end to the start codon. This is done separately for each read length (heatmap). **c** Examples of figures used to perform quality control on the data from a Ribo-seq experiment with four samples. Left column: The percentage of reads in each translation frame over CDSs after P-shifting, stratified by read-length. Right column: (1) Number of aligned reads after filtering contaminants. (2) Percentage of reads aligning to various transcript types (> 1%). (3) Percentage alignment of each transcript type in the "Other" group. (4) Percentage of reads aligning to mRNA falling into the CDS or UTRs. For more QC examples see Additional file 1: Figs. S5 and S6. **d** Read coverage tracks for scanning ribosomal small subunit (SSU), 80S translating ribosomes and CAGE-defined transcription start sites (TSS) for a model gene. The gene has two transcript isoforms with different TSSs and 5′UTRs. The second isoform harbors an additional uORF leading to translational repression through ribosome dissociation (uORF 1). ORFik can assist in detecting such differences in isoform through differential expression, visualization, and metrics

A typical workflow takes transcriptome/genome annotation and high-throughput sequencing data as input, processes these to make transcriptome-wide tracks (Fig. 2a), and use these to either make summary statistics for all genes or transcripts, or to characterize one or more specific transcriptomic regions. Regions can be of any type and are completely user-customizable, but typically consist of genes, 5′ UTRs, CDSs, uORFs, start codons, or similar. ORFik can then be used to calculate summary statistics and features for all candidate regions.

ORFik supports standard translation analysis: it can map reads from RNA-seq and ribo-seq, it performs trimming and P-site shifting of ribo-seq reads, quantifies ribosomal occupancy (Fig. 2b), characterizes ORFs, and creates a range of plots and predictions. In addition, it supports the analysis of translation initiation through TCP-seq, RCP-seq, and CAGE. It can quantify translation initiation through scanning efficiency and ribosome recruitment, and can correlate these with sequence elements. Overall, ORFik provides a toolbox of functions that is extremely versatile and enables the user to go far beyond standardized pipelines.

#### Obtaining and preprocessing data

The first step in ORFik is obtaining and preprocessing data (Fig. 2a). It automates direct download of datasets from the NGS repositories: SRA [26], ENA [27], and DRA [28], download of annotations (FASTA genome and GTF annotation) through a wrapper to biomartr [29], while also supporting local NGS datasets and annotations. The sequencing data can be automatically trimmed with fastp [30] with auto removal of adapter sequences or any user defined sequence. Adapter presets for the most common Illumina adapters are included (TruSeq, small RNA-seq and Nextera), but other sequencing platforms can be used by customizing adapter removal. Following this, ORFik can detect and remove any of the known contaminants (e.g., PhiX 174, rRNA, tRNA, non-coding RNAs) and finally align the reads using STAR [31].

#### Quality control

Post-alignment quality control is initiated by calling the function ORFikQC. ORFik outputs quality control plots and tables for comparisons between all runs in an experiment and the whole process is streamlined to be as fast and simple as possible for the user. Among others, plots of the meta coverage across all transcript regions and correlation plots between all pairs of samples (Fig. 2d; Additional file 1: Fig. S5) are generated. Furthermore, essential mapping statistics are calculated such as the fraction of reads mapping to important features, contaminants, and transcript regions (Fig. 2c).

#### Single-base resolution of transcription start sites

Many organisms have extensive variations in the use of isoforms between tissues and can often use a range of sites to initiate transcription. Since many analyses of translation depend on an accurate annotation of 5′UTRs, ORFik supports the use of CAGE data. CAGE is a high-throughput assay for the precise determination of transcription start sites (TSSs) at single-base pair resolution [32]. ORFik makes use of CAGE (or similar 5′ detection assays) to reannotate transcripts. In a typical workflow (Additional file 1: Fig. S7), ORFik identifies all CAGE peaks in promoter-proximal regions and assigns the largest CAGE peak as the TSS. This can be customized to only consider specific thresholds or exclude ambiguous TSSs that are close to the boundary of other genes.

#### Automatic read length determination

When performing ribo-seq it is customary to size-select a range of fragments that correspond to the size protected by a single ribosome. This is because not all isolated fragments necessarily originate from regions protected by ribosomes. Instead, these can be the product of other sources such as RNA structure, RNA binding proteins or incomplete digestion [33]. This filtering of sizes is typically performed first in the lab and then computationally. To identify which read lengths most likely originate from actual ribosome footprints (RFPs), ORFik identifies read lengths that display 3 nt periodicity over protein-coding regions, indicative of ribosome translocation (Fig. 2a; Additional file 1: Fig. S1). For each fragment length, we sum the 5′ ends of footprints mapping to the first 150 nucleotides in the CDSs of the top 10% of protein-coding genes ranked by coverage and keep lengths with at least 1000 reads. The resulting vectors are subject to discrete Fourier transform, and the fragment lengths whose highest amplitude corresponds to a period of 3 are considered to be bonafide RFPs (Additional file 1: Fig. S8). By default, all read lengths that lack this periodicity are filtered out [34].

#### Sub-codon resolution through P-shifting

Sequenced RFP reads span the whole region where the ribosome was situated. In many analyses, however, it is interesting to increase the resolution and determine exactly which codon the ribosome was processing when it was captured, the so-called P-site. Several methods of determining P-site location within footprints (P-shifting) have previously been developed [12, 23, 34, 35]. ORFik predicts the P-site offset per read length, taking inspiration from the Shoelaces algorithm [34]. To determine the P-site offset in the protected footprints, ORFik considers the distribution of the different read lengths over the translation initiation site (TIS) region. For each read length ORFik takes the 5′ end of all reads from all genes and sums these for each coordinate relative to the TIS. ORFik then performs a change point analysis to maximize the difference between an upstream and downstream window relative to the changepoint (Additional file 1: Fig. S9). This analysis results in the most probable offset to shift the 5′ ends of fragments protected by initiating ribosomes exactly at the TIS (Fig. 2b). The function can process any number of libraries and can supply log files and heatmaps over the start and stop codon used to verify that the P-site detection was correct (Additional file 1: Fig. S10) and users can manually override the suggested shifts. The user can choose between several formats, where the two default formats are wiggle format (wig) and ofst (for very fast loading into R).

### Analysis

After preprocessing the user creates an instance of the “experiment” class. This summarizes and provides information about the data and makes it possible to analyze, plot, or create features for any number of NGS libraries. Experiments contain all relevant metadata so that naming and grouping in plots can be performed automatically. It also specifies the correct annotation and genomic sequence files for the data, enabling automatic downloading of these. To facilitate sharing between researchers the experiment class is constructed to not contain any local information like file paths. This makes it easy to send a short single script to collaborators that can perfectly replicate the entire process starting from scratch and producing identical downstream analyses and plots.

#### ORFik extends GenomicRanges and GenomicFeatures to the transcriptome

To facilitate the analysis of any region in the transcriptome, ORFik extends the functionality of two Bioconductor core packages: GenomicRanges and GenomicFeatures [24]. The intention of the first package was to provide robust representation and facilitate manipulation of contiguous (non-spliced) genomic intervals. GenomicFeatures, on the other hand, aimed at extending this functionality to spliced ranges and more complicated annotations, like gene models. The high-speed functions for intra- and inter-ranges manipulation of GenomicRanges are, however, inconsistent or completely lacking for the spliced ranges. ORFik extends these packages with more than 50 new functions for spliced ranges and more effectively converts between genomic and transcriptomic coordinates (Additional file 1: Table S5). ORFik also supports fully customizable subsets of transcript regions and direct subsetting of among others start codons, stop codons, transcription start sites (TSS), translation initiation sites (TIS), and translation termination sites (TTS).

ORFik also contains functions to facilitate rapid calculation of read coverage over regions. Beyond basic coverage per nucleotide, ORFik supports different coverage summaries like read length or translation frame in ORFs (Additional file 1: Table S7). All coverage functions also support the input reads as collapsed reads (merged duplicated reads with a meta column describing the number of duplicates). This greatly speeds up calculations and reduces memory consumption, especially for short-read sequencing data characterized by high duplication level (e.g., ribo-seq).

#### Visualizing meta coverage

Metaplots are a useful way of visualizing read coverage over the same relative region across multiple transcripts. ORFik implements these plots through a generalized syntax used for intuitive one-line functions. All plots can be extended or edited as ggplot objects (R internal graphic objects [36]). Since meta coverage and heat maps can be represented in multiple ways that emphasize different features of the data ORFik provides 14 different data transformations for metaplots; more basic ones like the sum, mean and median, and more advanced transformations like z-score (number of reads mapped to position—mean reads of region / standard deviation over region), position-normalized (number of reads mapped to position / total number of reads mapped to region), or mRNA-seq normalized (number of reads mapped to position / mRNA-seq FPKM of the entire gene) (equations in Additional file 1: Table S7). It also provides filters to avoid bias from single occurrences such as extreme peaks caused by contaminants in the data (e.g., non-coding RNAs). ORFik can filter these peaks or whole transcripts, to make plots more representative of the majority of the regions.

#### Identifying and characterizing open reading frames

ORFs can be split into a hierarchy based on the available evidence: (1) sequence composition and presence in a transcript, (2) active translation, measured with ribo-seq, (3) peptide product detection (4) confirmation of function. ORFik addresses the two first levels of this hierarchy.

Obtaining all possible ORFs based on sequence is accomplished through a scan of user-provided FASTA sequences to find candidate ORFs. The search is efficiently implemented using the Knuth–Morris–Pratt [37] algorithm with binary search for in-frame start codons per stop codon (Additional file 1: Table S5). It supports circular prokaryotic genomes and fast direct mapping to genomic coordinates from transcript coordinates. The provided sequence data can be any user-provided files or biostrings objects but the most straightforward approach is to obtain these from ORFiks own data loading functions. After identification, ORFs can be saved (e.g., in BED format with color codes) and loaded into a genome browser for visualization.

If based purely on the sequence, the number of ORFs in most genomes is vast [38]. When searching for uORFs or novel genes it can therefore be advantageous to move to the second step of the ORF hierarchy and also consider the ribosomal occupancy of the novel region. However, when analyzing small regions, like putative uORFs, simply observing the presence of reads will often have low predictive power. This stems from two issues: The first is a sampling problem, in that ribosomes over a short region might be transient and simply not get sampled and sequenced unless the occupancy is particularly high. The second is the aforementioned problem that not all reads originate from ribosomes. A weak ribo-seq signal alone is therefore not conclusive as evidence for translation [39].

To address this, several metrics or features have been produced that quantify how RNA fragments that originate from ribosome protection behave over verified protein-coding regions. ORFik currently supports more than 30 of these metrics that have been previously described in the literature, by us and others (Additional file 1: Table S1). ORFik also provides a wrapper function computeFeatures that produces a complete matrix of all supported metrics with one row per ORF or region. This resulting output matrix can be used to characterize specific ORFs or as input to machine learning classifiers that can be used to predict novel functional ORFs. By using a set of verified ORFs (e.g., known protein-coding sequences) the user can construct a positive set and use the features learned from them to classify the set of new candidate ORFs. Alternatively, the matrix may be exported and provided as input to other tools.

#### Differential expression

**Fig. 3:**
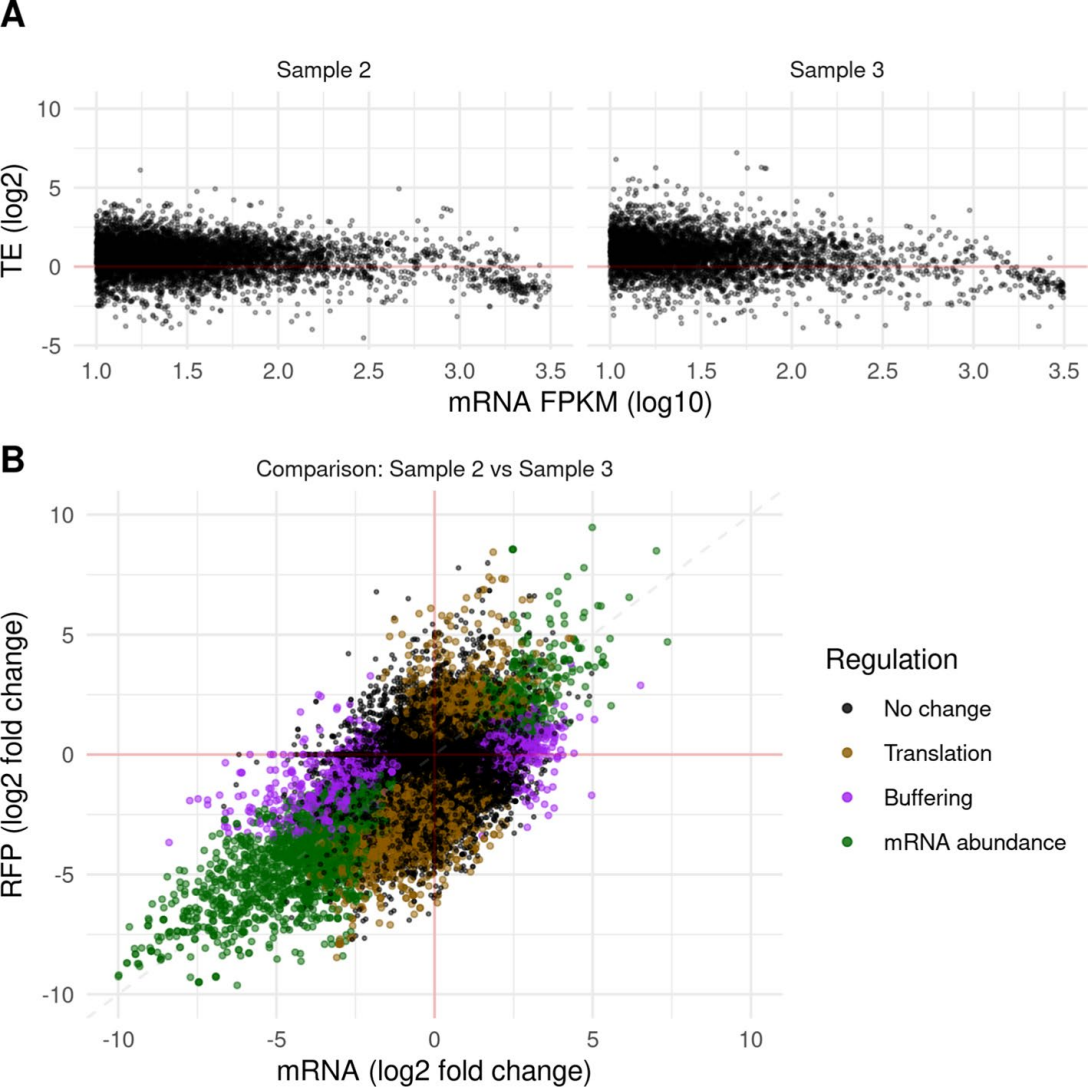
Analysis of differential regulation. ORFik supports within-group (**a**) and between-group (**b**) plots for analyzing translational regulation. **a** TE vs mRNA levels using the average values across replicates. **b** Differential translation analysis between conditions. Non-significant genes are colored black while significant genes (p < 0.05) are grouped into 3 categories according to deltaTE classification [40]: Translational regulation — mRNA abundance is static while translation changes (orange), Buffering — mRNA and ribosome profiling regulated in opposite directions (purple), mRNA abundance regulation — significant change in mRNA abundance and a corresponding significant change in ribo-seq levels (green). Example data from two zebrafish developmental stages from Bazzini et al. 2014 (Additional file 1: Table S9), each with two replicates [46]

ORFik supports various ways of estimating differential expression or preparing data for other tools. For visualization, it supports standard plots for studying fold changes of translation and RNA, and the relationship between expression and translational efficiency (Fig. 3a). Since many tools require raw counts as input for analysis of expression [40–43], ORFik can provide count data tables through the countTable function. This can optionally collapse and merge replicates or normalize data to FPKM values. These count tables can be supplied to DESeq2, anota2seq, and other tools to perform differential expression analysis [40, 44]. Alternatively, an implementation of the deltaTE algorithm [45] is also included in ORFik, which can detect translational regulation between conditions using a Wald test for statistical significance (Fig. 3b).

## Results

To illustrate the functionality of ORFik we show two use cases, where we (1) study translation initiation with profiling of scanning SSUs, and (2) make a simple pipeline to predict and characterize translated uORFs.

### Using ORFik to analyze translation initiation

**Fig. 4:**
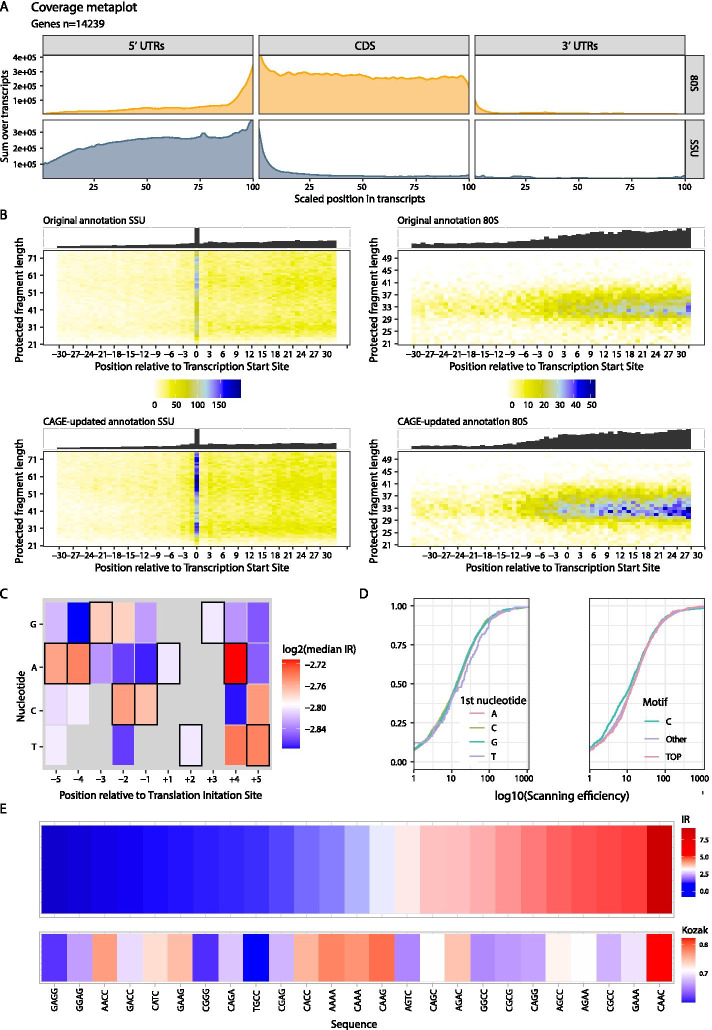
Analysis of translation initiation. **a** Meta coverage plot of RCP-seq data over 5′ UTRs, CDSs, and 3′ UTRs from all mRNA transcripts [8]. Regions are scaled to be the same size and coverage is displayed as the sum of reads for the translating (80S) and scanning ribosomes (SSU). **b** 5′ end coverage heatmaps of SSU reads (left column) and 80S (right column) from TCP-seq relative to transcription start site. Coverage is shown before (upper row) and after (lower row) reannotation of transcription start sites with CAGE data. **c** Analysis of initiation context showing the median initiation rate (IR) for translation initiation sites with a specific base (x-axis) at a specific position (y-axis). The strongest consensus is displayed with a black frame. **d** Analysis of ribosome recruitment showing the relationship between different TSS contexts and SSU scanning efficiency. Left: empirical cumulative distribution function (ecdf) of scanning efficiency colored by the first nucleotide in the 5′UTR. Right: ecdf of scanning efficiency for 3 different motifs; a C nucleotide, the TOP motif (C, then 4 pyrimidines), and all other TSS variants. **e** Initiation contexts ranked by IR. Top: mean IR for all CDSs with specified sequence (x-axis) relative to TIS (− 4 to − 1). Bottom: similarity score to human Kozak sequence as defined in [48, 49]

When analyzing data that includes small subunit profiling the goal is often to understand or characterize the regulation of translation initiation. ORFik was here applied to a TCP-seq data set from HeLa cells [8], and easily produces metaprofiles over transcripts, separated into scanning SSU and 80S elongating ribosomes (Fig. 4a). As expected, the SSU scanning complexes display the highest coverage in 5′UTRs, while elongating ribosomes are enriched over the CDSs. To obtain accurate measurements of scanning over 5′UTRs, CAGE data was used to reannotate the TSSs. The effect on the coverage of SSU complexes around the TSS as a result of this reannotation can be viewed in Fig. 4b. These heatmaps have been *transcript-normalized*, meaning that all the reads mapping to one transcript have been normalized to sum to 1. This has the effect of weighing each transcript equally, but ORFik supports a range of other normalization methods that emphasize different aspects of the data (Additional file 1: Fig. S11). The increased coverage and sharper delineation of the TSS that can be seen in these plots illustrate that CAGE-reannotation is important even in well-annotated transcriptomes like the human. Accurate TSS is also important when studying features at the start of the transcript such as the presence of a TOP motif [47]. TOP motifs are known to be involved in the regulation of specific genes and ORFik can correlate such features to other observations of the transcript. An example of such an analysis is associating motifs with the scanning efficiency (SE) — the number of scanning ribosomes on the 5′UTR relative to the RNA abundance [5]. In our latest study, we showed an association between ribosome recruitment and TOP motifs during early zebrafish development [6]. ORFik easily produces a similar analysis for the HeLa cells revealing no such relationship in these cells.

Beyond recruitment, an important part of translation initiation is the recognition of the start codon. This is facilitated by the context surrounding it and ORFik provides several ways of exploring these features. At the level of basic visualization, heatmaps similar to those for TSS can be produced to explore the conformations and periodicity around the start codon (Additional file 1: Fig. S10). Provided with small subunit profiling, the initiation rate (IR)—the number of 80S elongating ribosomes relative to SSU scanning [5] — can also be calculated. Together with the sequence, this allows for investigating which initiation sequences lead to the most productive elongation (Fig. 4d, e).

### Detecting translated upstream open reading frames

**Fig. 5:**
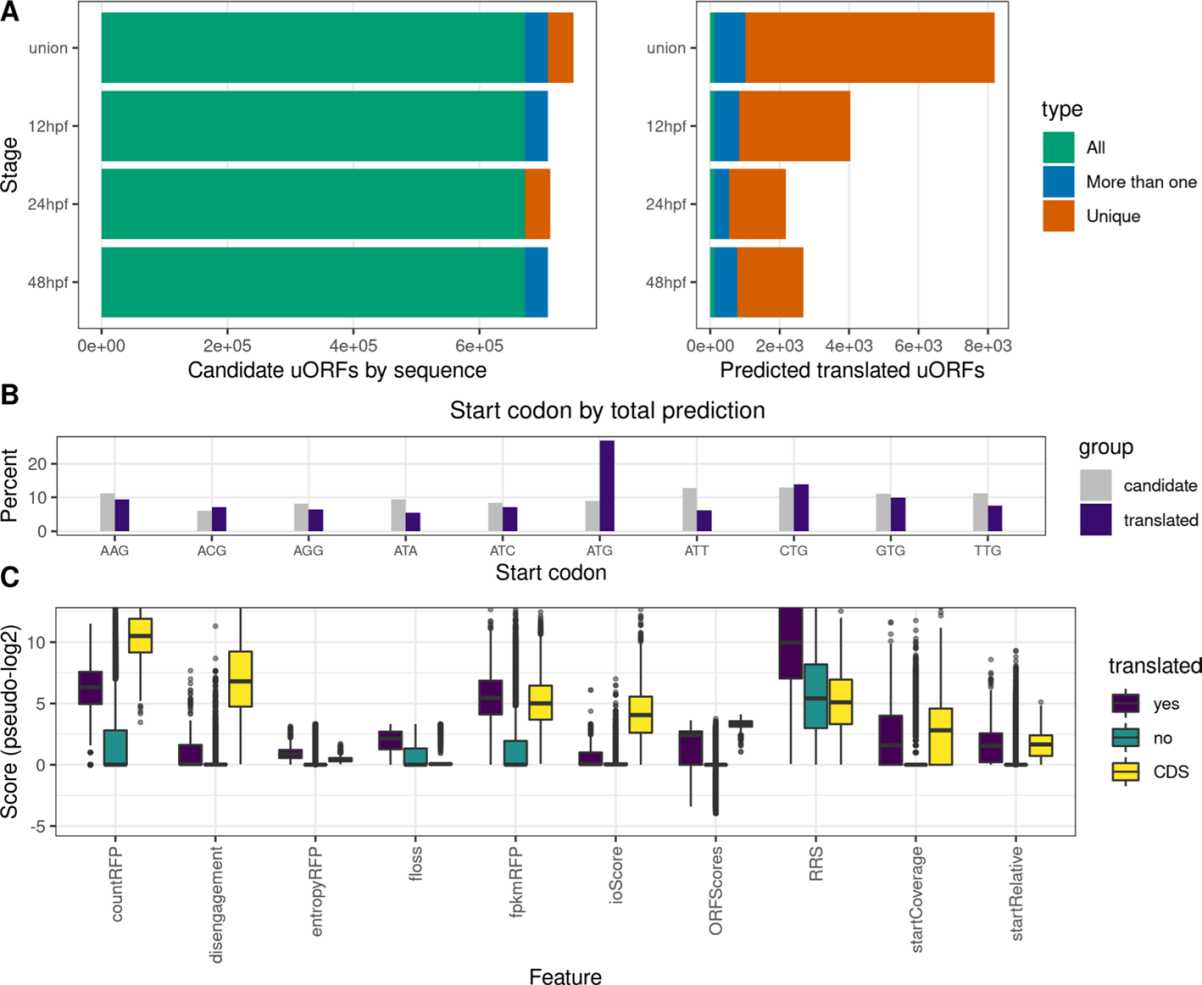
Prediction of uORFs in three zebrafish developmental stages. **a** Left panel: Number of candidate uORFs identified from sequence per stage. The union group describes the union set of all three timepoints. uORFs are colored whether they are common to all stages (green), shared between multiple (blue), or unique to one stage (brown). Right panel: Number of uORFs predicted to be actively translated. Direct comparison of overlapping uORFs between stages can be seen in Additional file 1: Fig. S12. **b** Distribution of start codons for candidate uORFs (gray) and predicted translated uORFs (purple). **c** Metrics used as features in classification (x-axis) shown for uORFs predicted to be translated (purple), predicted not to be translated (green), and protein-coding CDSs (yellow). See Additional file 1: Table S1 for a description of features. Y-axis pseudo-log2 score (abs(log2(score)) if score > 0.01, − log2(-score) if score < −0.01, and 0 if absolute value of score is < 0.01)

To illustrate an example of a search for novel translated regions, we applied ORFik to the problem of discovering differential uORF usage across three developmental stages of zebrafish embryogenesis: 12 h post-fertilization (hpf), 24 hpf, and 48 hpf [46]. Using public ribo-seq, RNA-seq [46], and CAGE [50] data we used ORFik to derive stage-specific 5′UTRs and searched these for all ATGs and near-cognate start codons. ORFik supports a number of features that have been shown to correlate with translation activity (Additional file 1: Table S1). We used ORFik to calculate all these metrics per stage for: (1) all uORF candidates, (2) all CDSs of known protein-coding genes, and (3) random regions from the 3′ UTRs. We used these to train a random forest model on each stage with the H2O R-package [51] using the CDSs as a positive set and the 3′UTR regions as the negative set (Additional file 1: Supplementary Note 1). This model was used to predict translational activity over the uORF candidates resulting in 8191 unique translated uORFs across all 3 stages (12hpf: 4042, 24hpf: 2178, 48hpf: 2676). Of these, only 133 are translated in all stages (Fig. 5a). Given the stringency of our training set which consists of long, translated proteins (positive), contrasted with short non-translated 3′UTR regions (negative) this should be considered a very conservative prediction. More nuanced predictions could be achieved by tuning these sets to better represent the typically shorter, more weakly translated uORFs.

For the predicted translated uORFs a clear difference in the usage of start codons can be seen relative to all possible candidate uORFs by sequence (Fig. 5b). While purely sequence-predicted uORFs show a relatively uniform distribution in start codon usage, a clear bias towards ATG followed by CTG can be seen for the translated uORFs. Since the classifier favors uORFs that start with start codons known to be strong initiators, despite not using start codon sequence as a feature, suggest that it is able to identify uORFs that are actively translated. Since the classifier is trained on CDSs, the resulting uORFs also have features that resemble known protein-coding genes (Fig. 5c).

## Summary

In summary, we have developed ORFik, a new API and toolkit for streamlining analysis of ORFs and translation. ORFik introduces hundreds of tested, documented, and optimized methods to analyze and visualize ribosome coverage over transcripts. It supports a range of data formats and can be used to create complete pipelines from read processing and mapping to publication-ready figures. We demonstrate its use on a transcriptome-wide study of translation initiation and quick annotation of translated uORFs. Together, this empowers users to perform complex translation analysis with less time spent on coding, allowing the user to focus on biological questions.

## Supplementary Information

**Additional file 1.** Supplementary file for ORFik: a comprehensive R toolkit for the analysis of translation

## Data Availability

Code for figures, tables and alignment of data: https://github.com/Roleren/ORFik_article_code. The datasets analysed during the current study will be downloaded automatically when running the scripts above. They can also be found in the respective sequence read archives referenced in Additional file 1: Table S9.

## Abbreviations

ORF: Open reading frame
CDS: Coding sequence (main ORF of mRNA)
5′ UTR: 5′ Untranslated region (also called transcript leader)
3′ UTR: 3′ Untranslated region (also called transcript trailer)
uORF: Upstream open reading frame
NGS: Next-generation sequencing
RFP: Ribosome-protected footprint (also referred to as RPF)
P-, E-, A-site: TRNA binding sites in the ribosome (peptidyl, exit and aminoacyl)
P-shifting: Relative 5′ end shifting of Ribo-seq reads to their estimated P-site
HPF: Hours post-fertilization
TSS: Transcription start site
TIS: Translation initiation site
SSU: Small subunit (ribosomal)
80S: 80S elongating ribosome

## Availability and requirements

**Project name**: *ORFik*

**Project home page**: http://bioconductor.org/packages/ORFik

**Archived version**: ORFik

**Operating system(s)**: Platform independent

**Programming language**: R

**Other requirements**: R version ≥ 3.6, Bioconductor version ≥ 3.10

**License**: MIT+ file LICENSE

**Any restrictions to use by non-academics**: none.
