# Supplementary material for: ORFik: a comprehensive R toolkit for the analysis of translation
Source: BMC Bioinformatics. 2021 Jun 19;22:336. doi: 10.1186/s12859-021-04254-w (PMC8214792; doi:10.1186/s12859-021-04254-w)
Supplement: Supplementary file 1 — Additional file 1. Supplementary file for ORFik: a comprehensive R toolkit for the analysis of translation [file 12859_2021_4254_MOESM1_ESM.docx]

# Supplementary material

[**Table S1**](#stabl_scoring)***: Scoring functions included in ORFik.*** *ORFik supports many published scoring functions for prediction of translated ORFs.* *The scoring functions column shows the names of the sequence and translation features used in the computeFeatures function and a few stand alone functions. Description column briefly describes each feature. Using ORFik back-end functions, you can easily create your own functions if needed.*

| **Scoring functions** | **Description** | **Published in** |
| --- | --- | --- |
| fpkm | Fragments per kilobase of exon per million reads (supports 4 different read normalizations, DEseq, total, overlapping and custom). | Trapnell et al. 2010 [[1]](https://paperpile.com/c/1KmOlc/OVcc1) |
| translationalEff | Translational efficiency. Ribo-seq / RNA-seq FPKM. You can normalize the libraries as in fpkm(). | Ingolia et al. 2009 [[2]](https://paperpile.com/c/1KmOlc/rc1AO) |
| floss | Fragment length similarity score. A statistic of how similar the observed ribo-seq read lengths is to those over verified ORFs. | Ingolia et al. 2014 [[3]](https://paperpile.com/c/1KmOlc/xnnIR) |
| entropy | Uniformity of read coverage across a region. | Ji et al. 2015 [[4]](https://paperpile.com/c/1KmOlc/tjram) |
| startRegionCoverage | Read count over translation initiation region. Region width defined by user. | Calviello et al. 2015 [[5]](https://paperpile.com/c/1KmOlc/jxToy) |
| startCodonCoverage | Read count over start codon. | Calviello et al. 2015 [[5]](https://paperpile.com/c/1KmOlc/jxToy) |
| startRegionRelative | Mean read count at nucleotides of the start codon relative to mean coverage of the surrounding region (-3 to 9). | ORFik |
| stopStallingScore | Read count over stop site / read count over whole ORF normalized by length. | Zhang et al. 2017 [[6]](https://paperpile.com/c/1KmOlc/MOqP3) |
| ribosomeReleaseScore (RRS) | Ratio of mean read count over ORF and 3’ UTR. | Guttman et al. 2013 [[7]](https://paperpile.com/c/1KmOlc/nnv43) |
| disengagementScore | Ratio of read coverage of ORF and downstream (to the end of transcript). | Chew et al. 2013 [[8]](https://paperpile.com/c/1KmOlc/ee7Wz) |
| insideOutsideORF | Ratio of read count in ORF relative to the rest of the transcript | Chew et al. 2013 [[8]](https://paperpile.com/c/1KmOlc/ee7Wz) |
| initiationScore | Difference in distribution of read lengths from ribo-seq over the translation initiation region of a candidate ORF relative to the distribution from all CDSs. | Giess et al. 2017 [[9]](https://paperpile.com/c/1KmOlc/zhJxN)  Calviello et al. 2015 [[5]](https://paperpile.com/c/1KmOlc/jxToy) |
| orfScore | Captures the distribution of reads across frames. The score is positive if the first frame has more reads than frame 2 and 3. Negative otherwise | Bazzini et al. 2014 [[10]](https://paperpile.com/c/1KmOlc/hAfZU) |
| findPeaksPerGene | Detects peaks based on z-score of each position relative to the rest of the ORF. | Kumari et al. 2018 [[11]](https://paperpile.com/c/1KmOlc/yiPRW) |
| kozakSequenceScore | Similarity of sequence of translation initiation site to Kozak reference. Supports multiple species, and user-specified sequences. | Grzegorski et al. 2014 [[12]](https://paperpile.com/c/1KmOlc/kGIOi) |
| isOverlapping | Is the candidate ORF overlapping any already verified ORFs (usually CDS). | ORFik |
| rankOrder | The 5’ position rank of the ORF (ORF closest to TSS is rank 1). | ORFik |
| fractionLength | Size of ORF relative to the whole transcript. | Chew et al. 2013 [[8]](https://paperpile.com/c/1KmOlc/ee7Wz) |
| distToTSS | Distance from ORF start site to transcription start site (TSS). | ORFik |
| distToCDS | Distance from verified CDS start site to candidate ORF stop site. | ORFik |
| isInFrame | Is ORF in the same frame as another ORF / CDS. | ORFik |
| stopCodonGrouping | Group all ORFs that share the same termination site. | ORFik |

[**Table S2**](#stabl_compare)***:*** ***Comparison of functionality from translation tools.*** *Figure updated and expanded from Ribotoolkit manuscript supplementary data (*Table S2*)* [*[13]*](https://paperpile.com/c/1KmOlc/kPCp)*.*


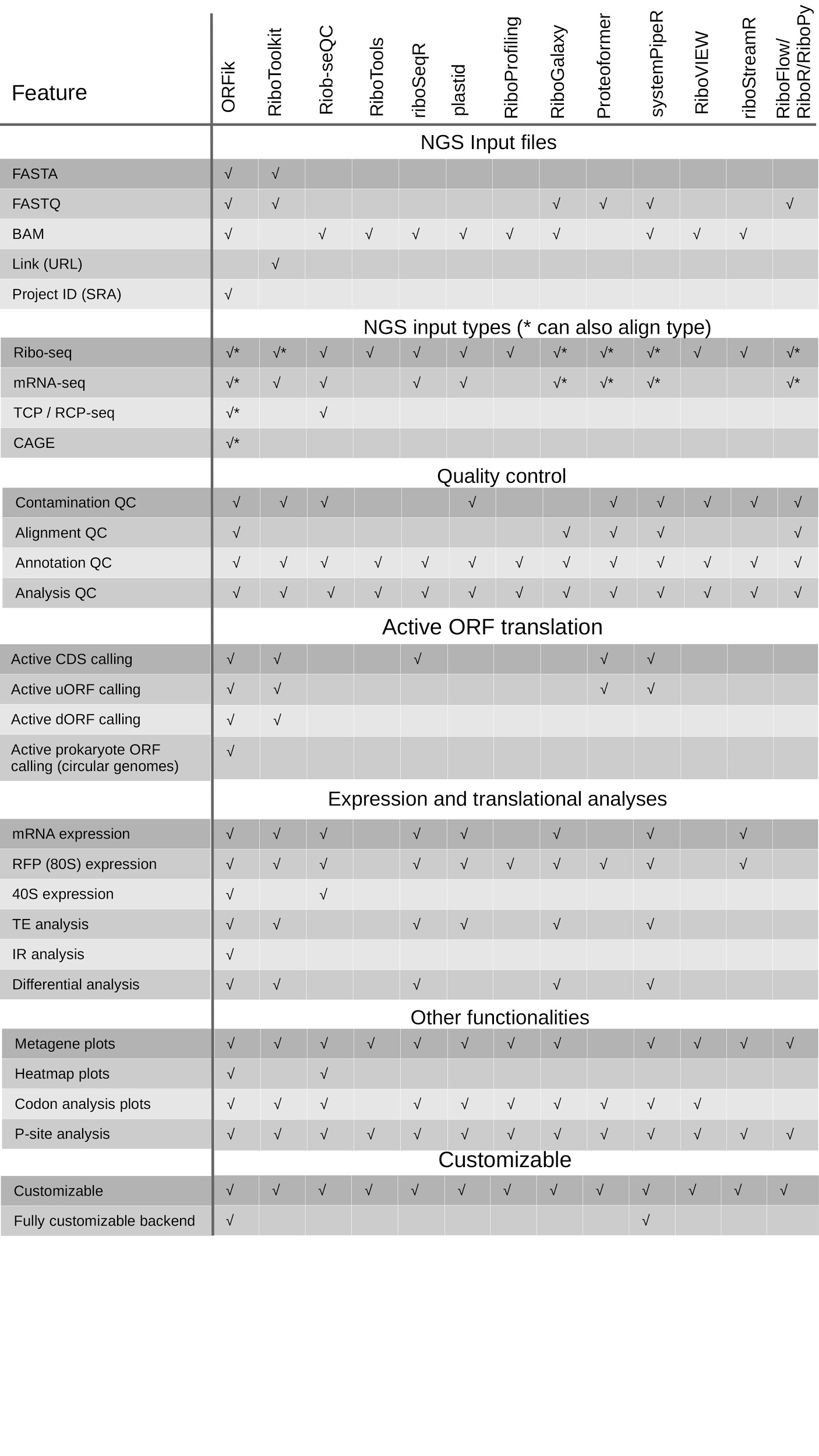


##

[**Table S3**](#stabl_pshiftdiff)***: Comparison of automatic P-site offset detection: ORFik, Ribotoolkit, Shoelaces and Riboprofiling.***

*Reads from highly abundant read-lengths (21,27,28) were used and shift offset was found with both programs. In cases where the offset difference between the programs is in-frame the impact on downstream analysis is lower as compared to out-of-frame shifts. A visual representation of these read lengths around the TIS can be found in Figure S2. Data used: Alexaki et al 2020 (*[Additional file 1: Table S9](#sta_data)*)* [*[14, 15]*](https://paperpile.com/c/1KmOlc/Bkfb+z31b)*. * Read length did not pass the threshold of Ribotoolkit P-shifting.*

| **Library** | **Length** | **Offset orfik** | **Offset ribotoolkit** | **Offset shoelaces** | **Offset Riboprofiling** | **All In Frame** |
| --- | --- | --- | --- | --- | --- | --- |
| CO1 | 21 | −12 | −3 | −9 | −3 | TRUE |
| CO1 | 27 | −12 | −11 | −9 | −3 | FALSE |
| CO1 | 28 | −12 | −12 | −12 | −6 | TRUE |
| CO2 | 21 | −12 | −3 | −9 | 0 | TRUE |
| CO2 | 27 | −12 | −12 | −9 | −3 | TRUE |
| CO2 | 28 | −12 | −12 | −12 | −6 | TRUE |
| CO3 | 21 | −12 | * | −9 | 0 | * |
| CO3 | 27 | −11 | −11 | −11 | −3 | FALSE |
| CO3 | 28 | −12 | −12 | −12 | −6 | TRUE |
| WT1 | 21 | −12 | −9 | −9 | 0 | TRUE |
| WT1 | 27 | −12 | −11 | −11 | −3 | FALSE |
| WT1 | 28 | −12 | −12 | −12 | −6 | TRUE |
| WT2 | 21 | −12 | −3 | −9 | −3 | TRUE |
| WT2 | 27 | −12 | −12 | −11 | −3 | FALSE |
| WT2 | 28 | −12 | −12 | −12 | −6 | TRUE |
| WT3 | 21 | −12 | −3 | −9 | −3 | TRUE |
| WT3 | 27 | −12 | −12 | −11 | −3 | FALSE |
| WT3 | 28 | −12 | −12 | −12 | −6 | TRUE |

[**Table S4**](#stabl_tediff)***: Overlap of differential translation prediction by ORFik (deltaTE), Riborex and anota2Seq on data from Bazzini et al data 2014 (***[Additional file 1: Table S9](#sta_data)***).*** *The differential translational efficiency genes (DTEG) is the number of significant genes found, DTEGs overlap (%) is the percentage of genes also found to be significant by ORFik. The DTEGs can be split into three distinct categories shown by the remaining columns including overlap with ORFik. The overlap for the ORFik row shows the overlap of ORFik with anota2seq (Riborex does not classify DTEG in categories). The total overlap of DTEGs for Riborex and anota2seq was 41.86% (not shown in table).*

| **Tool** | **DTEGs** | **DTEGs overlap** | **Translation** | **Translation overlap** | **mRNA**  **abundance** | **mRNA abundance overlap** | **Buffering** | **Buffering overlap** |
| --- | --- | --- | --- | --- | --- | --- | --- | --- |
| ORFik | 4134 | 56.46 | 2419 | 48.66 | 502 | 63.15 | 1213 | 27.04 |
| anota2seq | 4683 | 49.84 | 3212 | 36.64 | 672 | 47.17 | 799 | 41.05 |
| Riborex | 2573 | 49.67 | * | * | * | * | * | * |

[**Table S5**](#stabl_speed)***: Runtime comparison of ORFik versus other tools***

***A) Comparison of runtime for tools finding ORFs.***

***i)*** *Finding uORFs on all 5’ UTRs in zebrafish transcriptome (Ensembl Danio rerio GRCz10, using a total of 11,343 5’ UTRs).* ***ii)*** *Finding all ORFs on all transcripts in zebrafish transcriptome (using a total of 59876 transcripts)*

*Arguments given: minimum length ORF: 30 bases (ORFfinder’s minimum), strand: sense (+), start codons: ATG, stop codons: TAA, TAG, TGA. Longest ORF per stop codon: FALSE and longest ORF per transcript: FALSE.*

**Average time of 3 runs. **ORFfinder linux x64 desktop version 2020-03-11 13:30* [*[16]*](https://paperpile.com/c/1KmOlc/FvzAu)*.***It should be noted that* systemPipeR::predORF was not designed for large scale ORF detection. Version: 1.23.4. **** RiboCode: Version: 1.3.1a, does not support direct finding of ORFs on 5’ UTRs, only over entire transcripts (which does include the uORFs) [[17]](https://paperpile.com/c/1KmOlc/ulkpT).

***B) Comparison in runtime of genomic to transcript coordinate mapping***

*Mapping all transcripts to transcript coordinates (Ensembl Danio rerio GRCz10, using a total of 57,369 transcripts). Output of functions are here identical. *Average time of 3 runs. GenomicFeatures version: 1.42.1*

***C) Comparison in load time of .bam file vs .ofst using ORFik::fimport****. Three different file types that load identical information into R (except QNAMES which are lost when collapsing reads), showing the increase in speed for loading collapsed .ofst files as compared to bam files. File used: 2hpf sample of ribo-seq from Bazzini et al 2014* [*[10]*](https://paperpile.com/c/1KmOlc/hAfZU)*, see* [Additional file 1: Table S9](#sta_data)*.*

| **A** | **ORFik** | **ORFfinder (ncbi)**** | **systemPipeR***** | **RiboCode** |
| --- | --- | --- | --- | --- |
| **i) time*** | 0.510 seconds | 46.361 seconds | 509.659 seconds | **** |
| **ii) time*** | 52 seconds | 1679 seconds | > 24 hours | 241 seconds |

| **B** | **ORFik**  **(pmapToTranscriptF)** | **GenomicFeatures**  **(pmapToTranscripts)** |
| --- | --- | --- |
| **time*** | 0.967 seconds | 16.700 seconds |

| **C** | **ORFik (fimport)**  **(collapsed .ofst)** | **ORFik (fimport)**  **(uncollapsed .ofst)** | **GenomicAlignments**  **(readGAlignments) (.bam)** |
| --- | --- | --- | --- |
| **time**  **(size of file)** | 0.254 seconds  (6.4 MB) | 23.046 seconds  (348.6 MB) | 414.936 seconds  (6728.9 MB) |

##

[**Table S6**](#stabl_transcriptAPI)***: Low level Transcript ranges API.*** *This API extends the Genomic ranges API for a more complete toolbox for spliced ranges, coverage and NGS data. The 3 columns describe the function names in ORFik, the type of function and gives a short description.*

| ***Function*** | ***Type*** | ***Description*** |
| --- | --- | --- |
| *Transcript getters* | | |
| *widthPerGroup* | *Transcript getters* | *Get width per transcript* |
| *seqnamesPerGroup* | *Transcript getters* | *Get seqname per transcript* |
| *strandPerGroup* | *Transcript getters* | *Get strand per transcript* |
| *numExonsPerGroup* | *Transcript getters* | *Get number of exons per transcript* |
| *firstExonPerGroup* | *Transcript getters* | *Get 5’ exon per transcript* |
| *lastExonPerGroup* | *Transcript getters* | *Get 3’ exon per transcript* |
| *firstStartPerGroup* | *Transcript getters* | *Get 5’ exon's 5’ start nt* |
| *firstEndPerGroup* | *Transcript getters* | *Get 5’ exon's 3’ start nt* |
| *lastExonStartPerGroup* | *Transcript getters* | *Get 3’ exon's 5 end nt* |
| *lastExonEndPerGroup* | *Transcript getters* | *Get 3’ exon's 3’ end nt* |
| *groupings* | *Transcript getters* | *Iterator for exons per transcript* |
| *start sites* | *Transcript getters* | *Get most upstream base, relative to strand* |
| *stop sites* | *Transcript getters* | *Get most downstream base, relative to strand* |
| *start codons* | *Transcript getters* | *Get 3 first 5’ bases of transcript, might be from more than 1 exon* |
| *stop codons* | *Transcript getters* | *Get 3 last 3’ bases of transcript, might be from more than 1 exon* |
| *startRegion* | *Transcript getters* | *Get custom region around start site* |
| *stopRegion* | *Transcript getters* | *Get custom region around stop site* |
| *numCodons* | *Transcript getters* | *widthPerGroup / 3* |
| *Range transformations* | | |
| *pmapToTranscriptF* | *Range transformations* | *Map from genomic to transcript coordinates* |
| *pmapFromTranscriptF* | *Range transformations* | *Map from transcript to genomic coordinates* |
| *txSeqsFromFa* | *Range transformations* | *Get DNA sequences from transcript coordinates* |
| *tile1* | *Range transformations* | *Tile transcript ranges to 1 nt ranges* |
| *downstreamN* | *Range transformations* | *Tile window of size N of transcript ranges to 1 nt ranges* |
| *windowPerGroup* | *Range transformations* | *Subset transcript to upstream & downstream range specified* |
| *Transcript setters* | | |
| *assignFirstExonsStartSite* | *Transcript setters* | *Assign 5’ exon's 5’ site* |
| *assignLastExonsStopSite* | *Transcript setters* | *Assign 3’ exon's 3’ site* |
| *downstreamOfPerGroup* | *Transcript setters* | *Extend downstream to point (which is excluded)* |
| *downstreamFromPerGroup* | *Transcript setters* | *Extend downstream to point (which is included)* |
| *upstreamOfPerGroup* | *Transcript setters* | *Extend upstream to point (which is excluded)* |
| *upstreamFromPerGroup* | *Transcript setters* | *Extend upstream to point (which is included)* |
| *extendLeaders* | *Transcript setters* | *Extend upstream to point (which is included), keep exon structure* |
| *extendTrailers* | *Transcript setters* | *Extend downstream to point (which is included), keep exon structure* |
| *Utility* | | |
| *sortPerGroup* | *Utility* | *Sort exons 5’ to 3’, relative to strand* |
| *removeMetaCols* | *Utility* | *Remove meta information* |
| *reduceKeepAttr* | *Utility* | *Remove exons of width 0 and bind connecting exons* |
| *unlistGrl* | *Utility* | *Transcript to exon grouping* |
| *groupGRangesBy* | *Utility* | *Exon to transcript grouping* |
| *orfID* | *Utility* | *Make a unique hash string per transcript from genomic range* |
| *uniqueGroups* | *Utility* | *Using orfID remove duplicated transcripts, from different isoforms / genes* |
| *export.bed12* | *Utility* | *Export transcript ranges with splicing information* |
| *NGS getters* | | |
| *fimport* | *NGS getters* | *Import NGS data, multiformat* |
| *readWidths* | *NGS getters* | *Get read widths* |
| *countOverlapsW* | *NGS getters* | *Count overlaps with weights* |
| *getWeights* | *NGS getters* | *Get weights of NGS reads* |
| *Coverage* | | |
| *coverageByTranscriptW* | *Coverage* | *Coverage per nt with weights, giving considerable speedup for coverage* |
| *coveragePerTiling* | *Coverage* | *Coverage per nt with weights, for plotting* |
| *scaledWindowPositions* | *Coverage* | *Binned Coverage, bin coverage of all transcripts to same length* |
| *metaWindow* | *Coverage* | *Coverage per nt in defined region* |
| *windowPerTranscript* | *Coverage* | *Coverage per region per nt* |
| *coverageScorings* | *Coverage* | *Coverage transformations* |
| *regionPerReadLength* | *Coverage* | *Coverage per transcript per read length per nt* |
| *windowPerReadLength* | *Coverage* | *Coverage per window per read length per nt* |
| *codonSumsPerGroup* | *Coverage* | *Coverage per codon* |

[**Table S7**](#stabl_transform)***: Coverage transformations in ORFik.*** *The coverage transformation available in ORFik through the function coverageScorings. This function can calculate meta coverage – summarizing and normalizing coverage over multiple regions. It takes as input the coverage/read count per position per transcript per library, optionally grouped by read length or transcriptomic feature (e.g. 5’ UTRs, CDS, 3’ UTRs). The output is either a normalization of the data, a grouping or a combination of these two. In the description column* ***x*** *is defined as the read count, r is a user-defined region (could e.g. be the neighborhood of all translation initiation sites, all uORFs or all 5’UTRs), p is the relative position within this region, f is a translation read frame, l is read length and n is the number of regions.*

| **Coverage transformations** | **Description:** | **Equation** |
| --- | --- | --- |
| sum | Sum of read counts x at position p over all regions. | [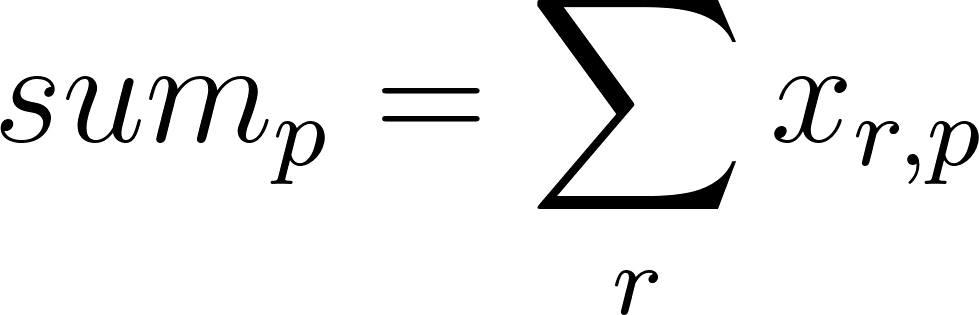](https://www.codecogs.com/eqnedit.php?latex=%20sum_p%20%3D%20%5Csum_r%20x_%7Br%2Cp%7D%20#0) |
| mean | Mean read count per position p over all regions. | [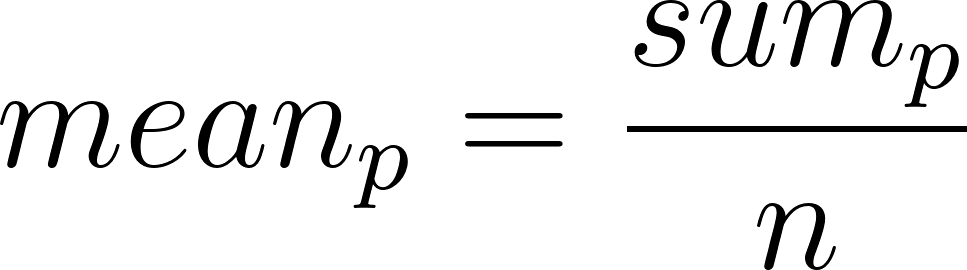](https://www.codecogs.com/eqnedit.php?latex=%20mean_p%20%3D%20%5Cdfrac%7Bsum_p%7D%7Bn%7D%20#0) |
| median | Median read count at position p over all regions. | [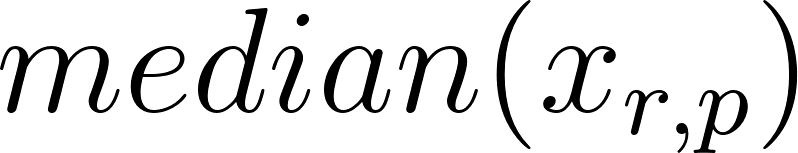](https://www.codecogs.com/eqnedit.php?latex=%C2%A0median(x_%7Br%2Cp%7D)%20#0) |
| fracPos | Fraction of read counts x at position p relative to all counts in one region r. | [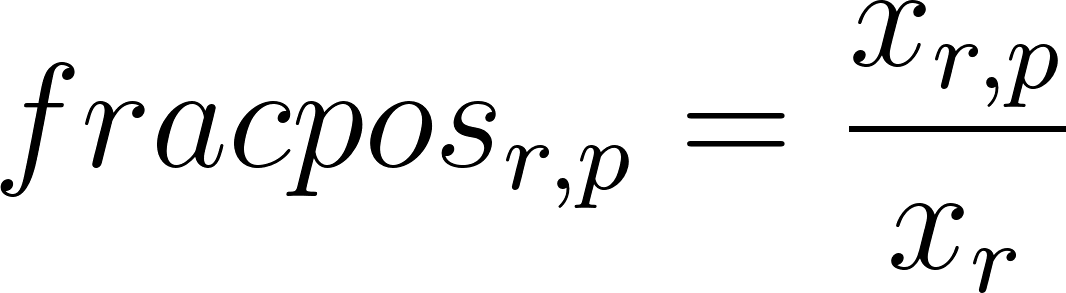](https://www.codecogs.com/eqnedit.php?latex=%20fracpos_%7Br%2Cp%7D%20%3D%20%5Cdfrac%7Bx_%7Br%2Cp%7D%7D%7Bx_%7Br%7D%20#0) |
| transcriptNormalized | Counts are normalized with fracPos so all counts per region sums to 1. These normalized values are summed over all regions for each position p. | [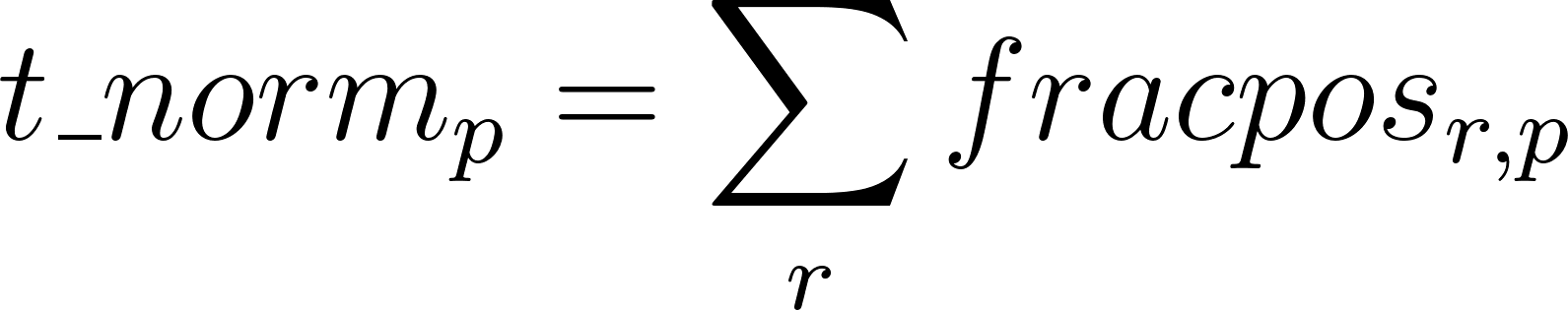](https://www.codecogs.com/eqnedit.php?latex=%20t%5C_norm_%7Bp%7D%20%3D%20%5Csum_r%20fracpos_%7Br%2Cp%7D%20#0) |
| zscore | Read count at each position is normalized relative to the distribution of read counts in the whole region. s is defined as standard deviation of read counts. | [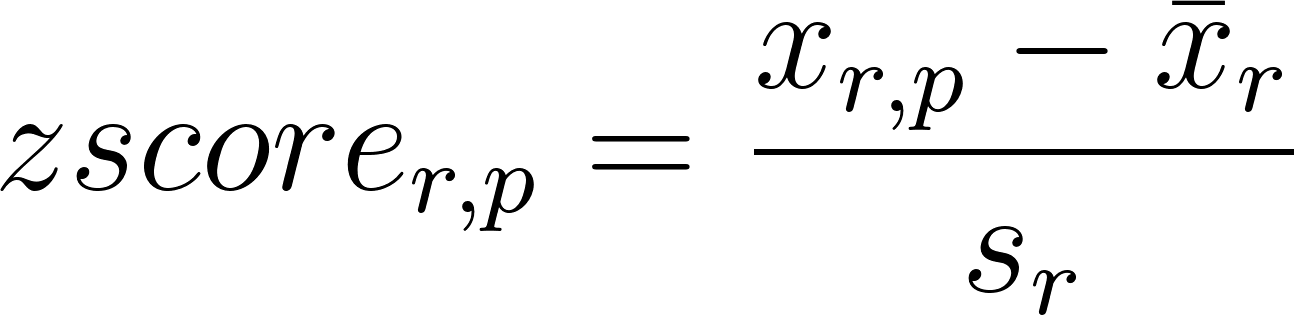](https://www.codecogs.com/eqnedit.php?latex=%20zscore_%7Br%2Cp%7D%20%3D%20%5Cdfrac%7Bx_%7Br%2Cp%7D%20-%20%5Cbar%7Bx%7D_r%7D%7Bs_r%7D%20#0) |
| log2sum | log base 2 of the read counts summed over all regions. | [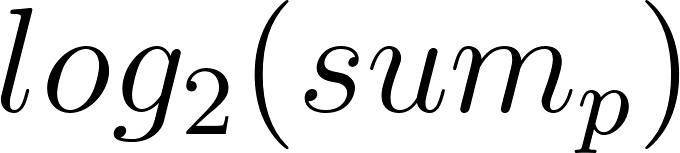](https://www.codecogs.com/eqnedit.php?latex=%20log_2%20(sum_p)%20#0) |
| log10sum | log base 10 of the read counts summed over all regions. | [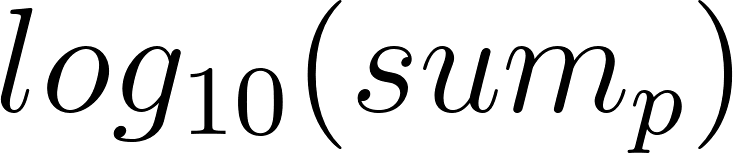](https://www.codecogs.com/eqnedit.php?latex=%20log_%7B10%7D%20(sum_p)%20#0) |
| sumPos | Sum of read counts x per binned window b in region r. b is here defined as a bin of at least one, but potentially multiple sequential nucleotides. A bin can be of a fixed size or relative to the size of r. | [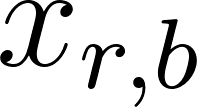](https://www.codecogs.com/eqnedit.php?latex=%20%20%20x_%7Br%2Cb%7D%20#0) |
| meanPos | Mean of read counts x per binned window b. b is here defined as a bin of at least one, but potentially multiple sequential nucleotides. \|b\| is the size of the bin in region r (can vary between regions for relative sizes). | [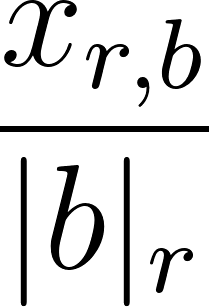](https://www.codecogs.com/eqnedit.php?latex=%20%20%5Cdfrac%7Bx_%7Br%2Cb%7D%7D%7B%7Cb%7C_r%7D%20#0) |
| frameSum | Sum of read counts x for each of the 3 translation frames f across all regions. | [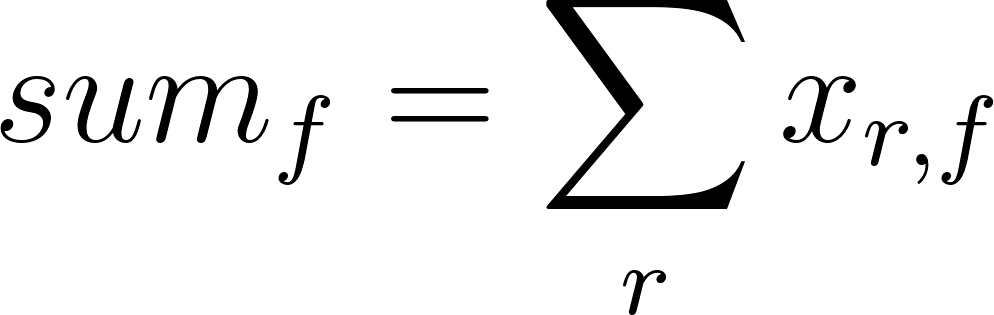](https://www.codecogs.com/eqnedit.php?latex=%20sum_f%20%3D%20%5Csum_r%20x_%7Br%2Cf%7D%20#0) |
| frameSumPerLG | Sum of read counts x in a set of regions r per frame f and read length l. Returns a count for each read length and frame for each of the regions r. | [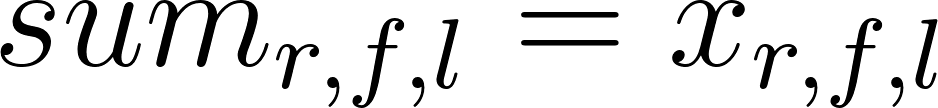](https://www.codecogs.com/eqnedit.php?latex=%20sum_%7Br%2Cf%2Cl%7D%20%3D%20x_%7Br%2Cf%2Cl%7D%20#0) |
| frameSumPerL | Sum of read counts x across all regions in a single translation frame f and for a single read length l. | [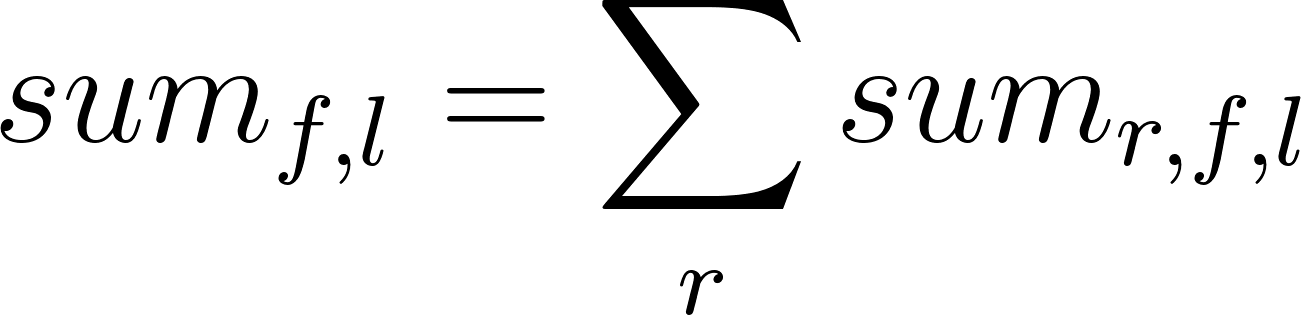](https://www.codecogs.com/eqnedit.php?latex=%20sum_%7Bf%2Cl%7D%20%3D%20%5Csum_r%20sum_%7Br%2Cf%2Cl%7D%20#0) |
| periodic | Does x show a periodicity of 3: TRUE or FALSE. | [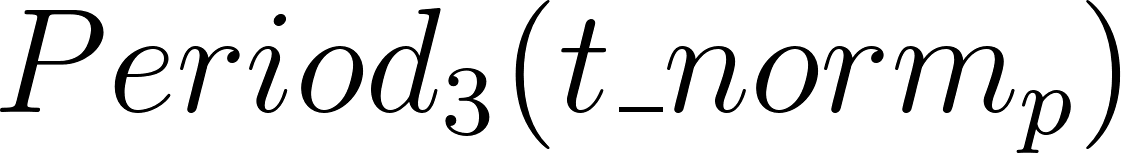](https://www.codecogs.com/eqnedit.php?latex=%20%20Period_3(t%5C_norm_p)#0) |
| NULL | No transformation |  |

[**Table S8**](#stabl_h20)***: H2O random forest prediction model statistics. A:*** *Consistency over repeated runs.* To show that the pipeline’s non-deterministic training step does not significantly impact the result, the uORFs from zebrafish development-stages were run through the pipeline 3 times, shown here in true positive rate and true negative rate. Giving the number of matched predicted true (translated) and negative (not translated) between the 3 runs. **B:** 10-fold cross validation to determine training model statistics. Shown are average accuracy, precision and recall of random forest training models over all 3 zebrafish development-stages.

| **A: Scoring feature** | **Description:** |
| --- | --- |
| true positive rate (match in predicted positives) | 98.1% |
| true negative rate (match in predicted negatives) | 99.5 % |
| **B: Scoring feature** | **Description:** |
| accuracy (mean +/- sd) | 0.9974 +/- 0.0012 |
| precision (mean +/- sd) | 0.9753 +/- 0.0102 |
| recall (mean +/- sd) | 0.9878 +/- 0.0073 |
| prediction type | Binary class prediction |
| number of trees (training) | 100 |

[**Table S9**](#stabl_data)***: Data used in this manuscript.*** *RNA-seq, ribo-seq and RCP-seq data was trimmed with fastp and aligned with STAR using ORFik wrapper. See the ORFik STAR and fastp script for the default arguments not specified here. For full details of data used see the attached alignment scripts.*

| **Feature** | **Ribo-**  **seq** | **RNA-**  **seq** | **CAGE** | **TCP-seq** | **CAGE** | **Ribo-**  **seq** | **RNA-**  **seq** |
| --- | --- | --- | --- | --- | --- | --- | --- |
| **Reference** | Bazzini et al  2014 [[10]](https://paperpile.com/c/1KmOlc/hAfZU)  (GSE53693) | Bazzini et al  2014 [[10]](https://paperpile.com/c/1KmOlc/hAfZU)  (GSE53693) | Nepal et al 2013 [[18]](https://paperpile.com/c/1KmOlc/oOrWJ) (SRA055273) | Bohlen et al  2020 [[19]](https://paperpile.com/c/1KmOlc/TlsO6)  (GSE139132) | Forrest et al, FANTOM5 Consortium  2014 [[20]](https://paperpile.com/c/1KmOlc/6ciU5)  (*DRR041459)* | Alexaki et al  2020 [[15]](https://paperpile.com/c/1KmOlc/z31b)  (PRJNA591214) | Alexaki et al  2020 [[15]](https://paperpile.com/c/1KmOlc/z31b)  (PRJNA591214) |
| **Species** | Danio rerio | Danio rerio | Danio rerio | Homo sapiens | Homo sapiens | Homo sapiens | Homo sapiens |
| **Genome-**  **annotation** | *Danio_rerio.GRCz10 (ensembl)* | *Danio_rerio.GRCz*  *10 (ensembl)* | *Danio_rerio.GRCz10 (ensembl)* | GRCh38.101 (ensembl) | GRCh38.101 (ensembl) | GRCh38.101 (ensembl) | GRCh38.101 (ensembl) |
| **Alignment, run mode:** | single end | single end | single end | single end | single end | single end | single end |
| **Adapter sequence trimmed** | AGATCGGAAGAGC | AGATCGGAAGAGC | - | AGATCGGAAGAGC | TGGAATTCTCGG | AGATCGGAAGAGC | AGATCGGAAGAGC |
| **3’ end bases trimmed** | 0 | 3 | 1 | 0 | 0 | 0 | 0 |
| **minimum length** | 20 | 20 | 20 | 20 | 20 | 20 | 20 |

## Supplementary Figures


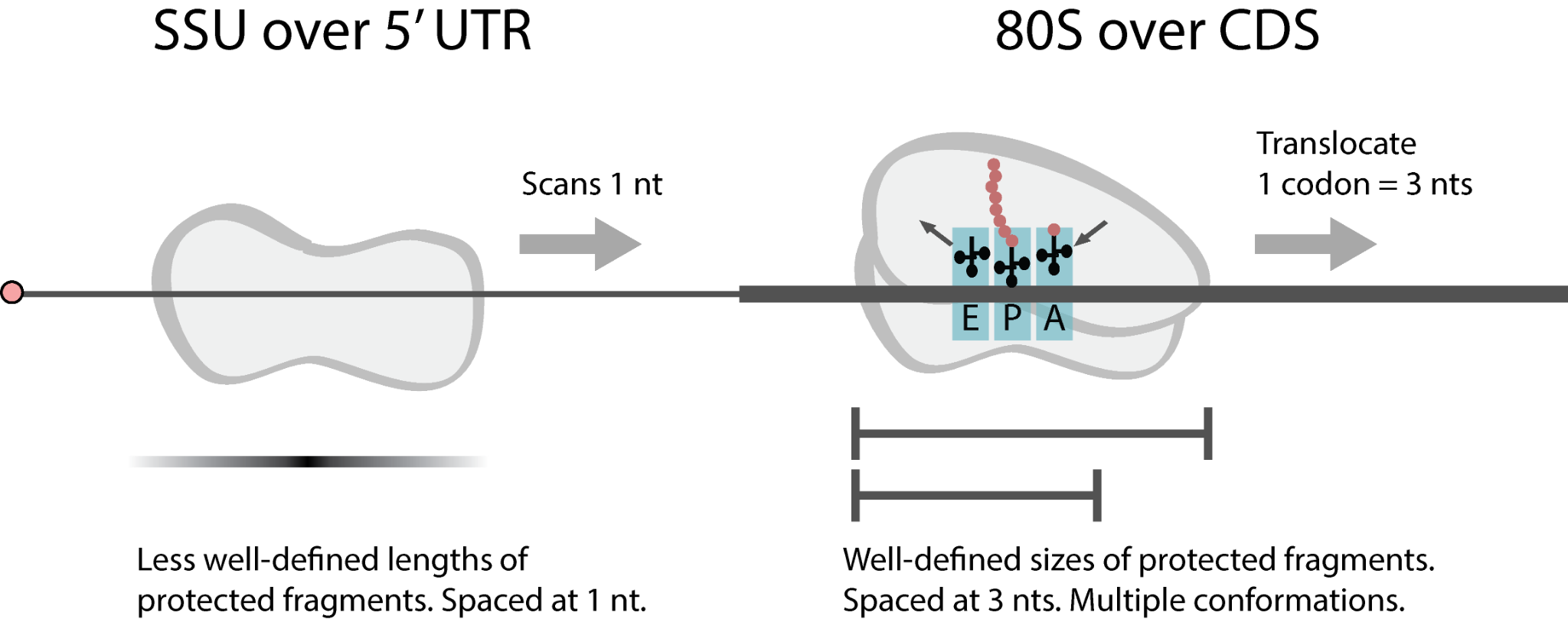


[**Figure S1**](#sfigu_spacing)***: Ribosomal scanning and translocation.*** *The small subunit (SSU) of the ribosome moves in 1 nt steps while scanning for an initiation site. The SSU protected fragments captured by TCP-seq/RCP-seq have shown diversity in the length of each fragment. The fragment lengths of 80S are more well-defined in a species-specific and protocol-dependent range. The 80S translocation in 3 nt = 1 codon steps results in a periodic spacing of protected fragments.*

*
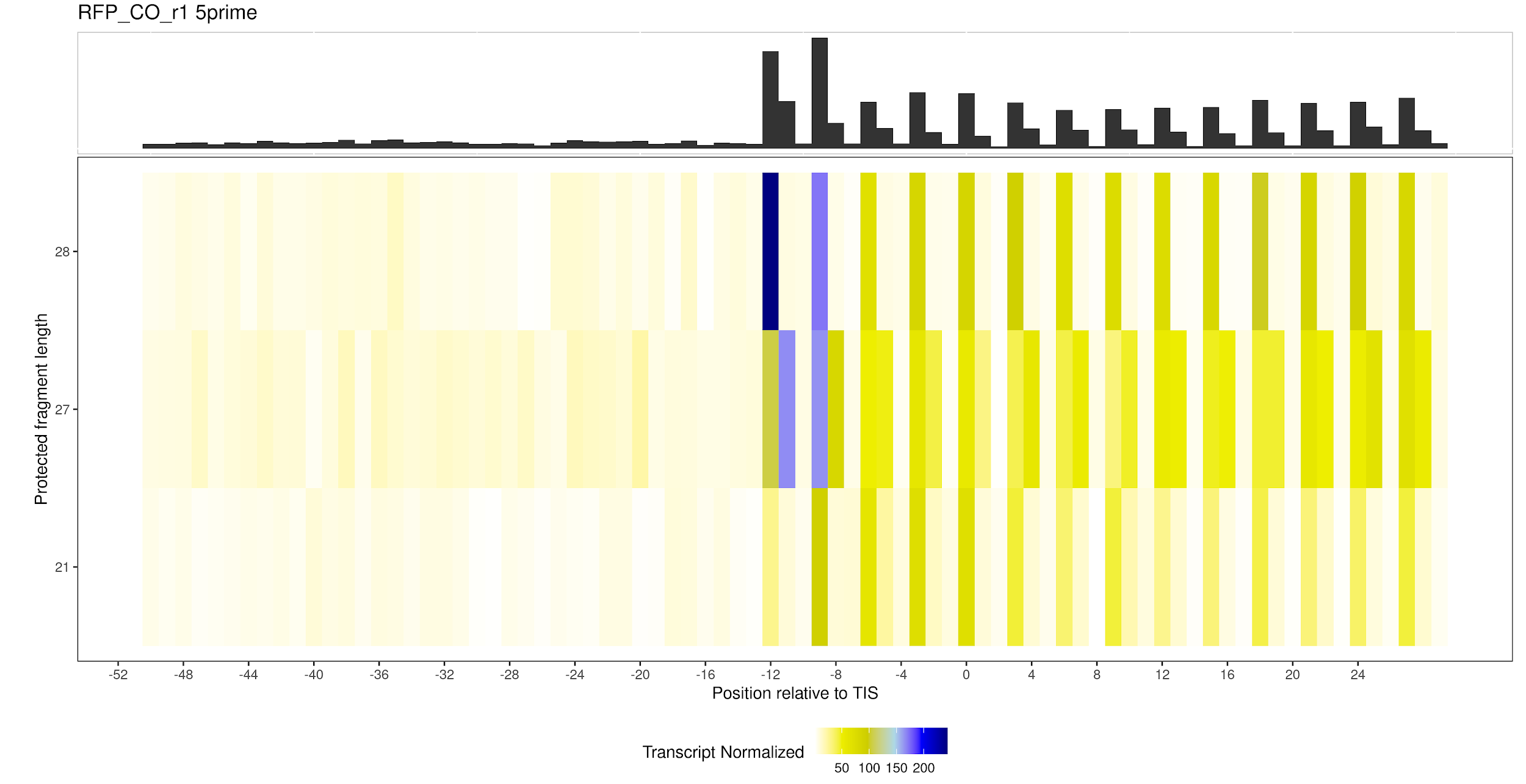
*

[**Figure S2**](#sfigu_benchheatmap)***: Ribo-seq coverage heatmap (before P-shifting)***

*Counts of 5’ end of reads from ribosome protected fragments around the translation initiation sites from the CO1 library in* [Additional file 1: Table S3](#sta_pshiftdiff) *(top 3 rows). Counts are stratified by their lengths (y-axis) and position relative to the TIS (x-axis, -52 to +29). The colors show transcript-normalized counts (all counts per transcript window sums to 1). Based on visual inspection, for read length 28 the best offset is likely -12 predicted by 3 out of 4 benchmarked tools. For length 27 it is unclear if the offset should be -12 or -11 (the tools do not agree). For read length 21 all tools agree on the frame, but different values (-12, -9, -3 and -3). Data from Alexaki et al 2020 (*[Additional file 1: Table S9](#sta_data)*), library: CO replicate 1* [*[15]*](https://paperpile.com/c/1KmOlc/z31b)*.*


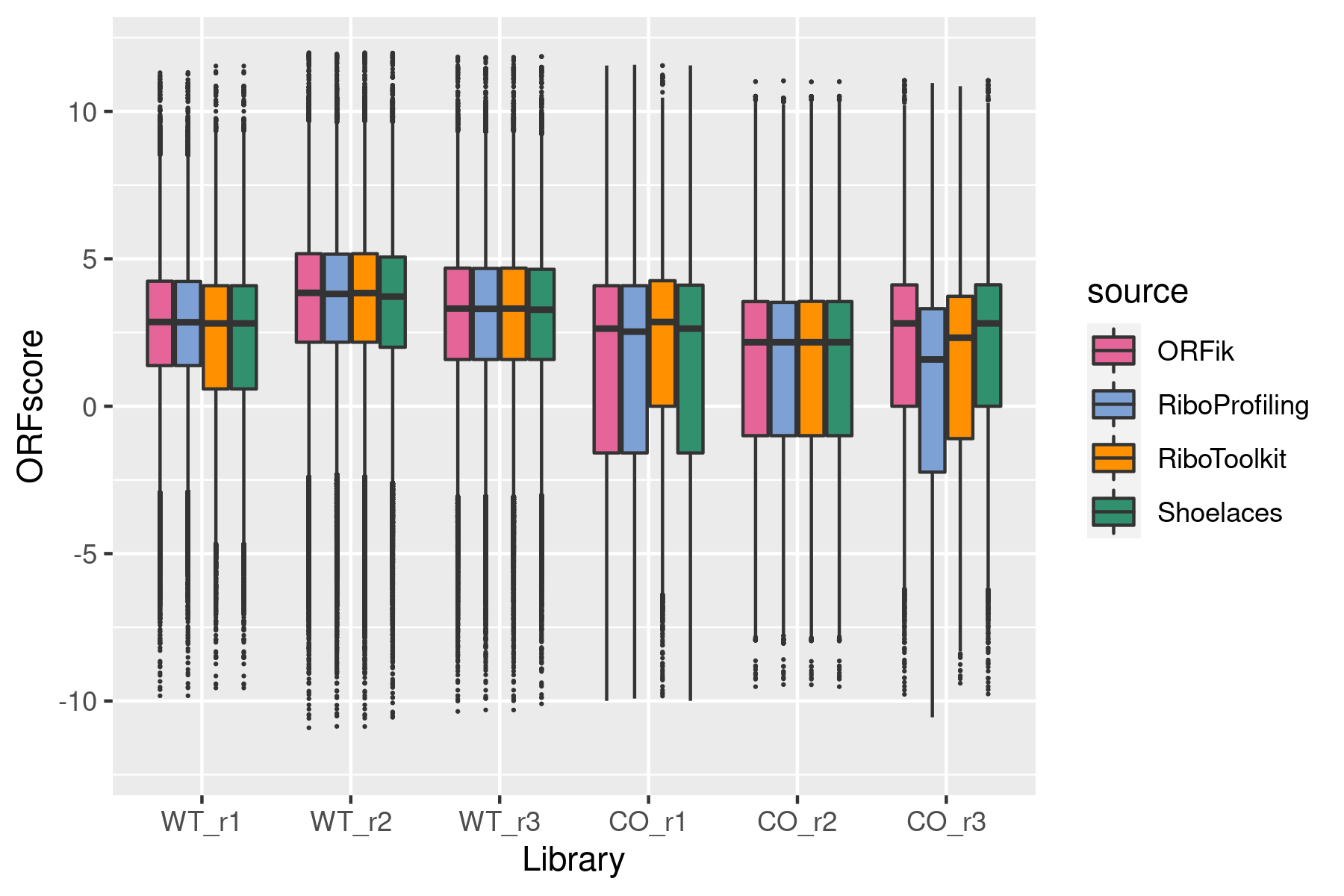


[**Figure S3**](#sfigu_orfscore)***: Benchmark comparison of P-shifting: ORFscore of ORFik, RiboProfiling, RiboToolkit and Shoelaces.***

*All 6 libraries of Ribo-seq from Alexaki et al 2020 were separately P-shifted with ORFik, RiboProfiling, Shoelaces and RiboToolkit (*[Additional file 1: Table S3](#sta_pshiftdiff)*, see alignment script for processing details)* [*[15]*](https://paperpile.com/c/1KmOlc/z31b)*. The strength of the 3 nt periodicity over all CDS with FPKM > 1 in all 6 libraries (45K CDSs) was determined using the ORFscore metric* [*[10]*](https://paperpile.com/c/1KmOlc/hAfZU)*. An ORFscore < 0 means either frame 1 or 2 have a stronger periodic coverage than the coding frame. In addition to the full length CDSs shown above, the tools were also run on a set of truncated CDSs, where 15 bases are removed from both 5’ and 3’ ends of each CDS. This is a common procedure to remove potential bias of reads proximal to the TIS and TTS regions. The ORFscores from these truncated CDSs were nearly identical, and with no significant change, to the full length (data not shown).*


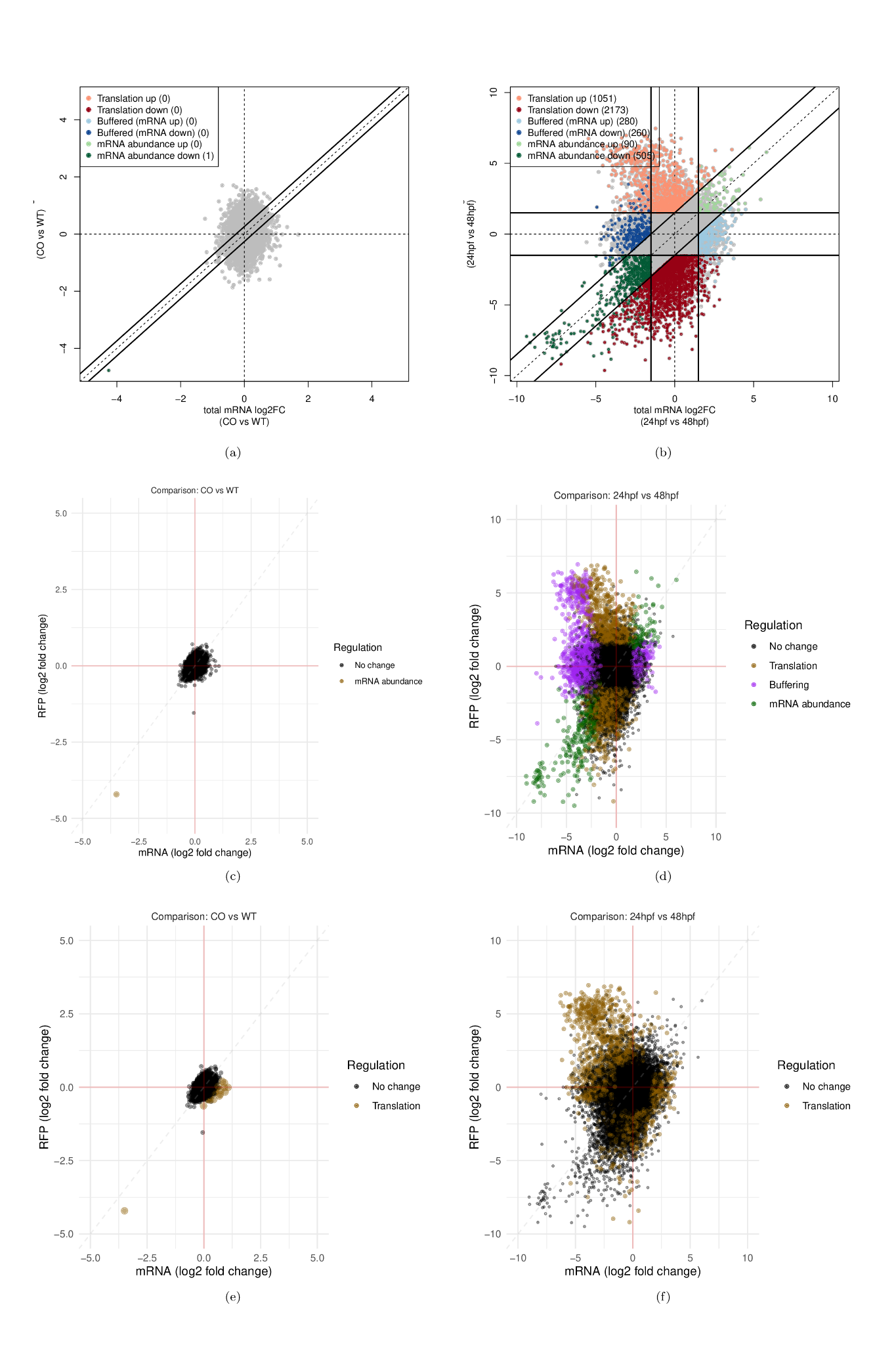


[**Figure S4**](#sfigu_benchTE)***: Comparison of differential translation analysis packages***

*Three comparisons of differential translation regulation analysis between anota2seq (a & b), ORFik (c & d) using the deltaTE algorithm* [*[21]*](https://paperpile.com/c/1KmOlc/y1XNu) *and Riborex (e & f)* [*[22]*](https://paperpile.com/c/1KmOlc/WaB7W)*. Each dot represents a single gene plotted as fold change of RNA-seq (x-axis) and ribo-seq (y-axis). For ORFik,* *the genes are grouped into four categories described in* [Figure 3](#fig_teMain)*. In addition, anota2seq also distinguishes between up and down regulation of the three significant categories. Riborex can not produce DTEG plots, so the results from Riborex are plotted using visualization functions from ORFik. It also does not classify DTEGs into categories, so they are here all classified as Translation.*

***False positive test: a), c) & e)*** *This figure illustrates that both ORFik and anota2seq*  *avoid****s*** *false positives in a set where only 1 gene has significant change. Riborex also detects this gene (F9), but in addition detects 19 additional genes as DTEGs (they have a higher TE log-fold change than F9). All six libraries of Alexaki et al 2020 were used: three replicates of WT and three replicates of CO (codon optimized F9 gene variant)* [*[15]*](https://paperpile.com/c/1KmOlc/z31b)*. The analysis was run on the longest transcript isoform per gene in ORFik****,*** *Riborex and anota2seq using a cutoff of p adjusted value < 0.05 with Benjamini-Hochberg correction.*

***Predicted group overlap test b), d) & f):*** *The test checked the amount of overlap in prediction of the algorithms in a set where many genes are expected to change. Analysis between 24 hour post fertilization and 48 hours post fertilization of Bazzini et al 2014 (two replicates per group). The analysis was run on the longest transcript isoform per gene in ORFik****,*** *Riborex and anota2seq using a cutoff of p adjusted value < 0.1 and absolute value log fold change > 1.5 with Benjamini-Hochberg correction. A clear trend is shown in that anota2seq is more likely to categorize genes into translation compared to the ORFik deltaTE implementation which categorizes more into Buffering.*

*
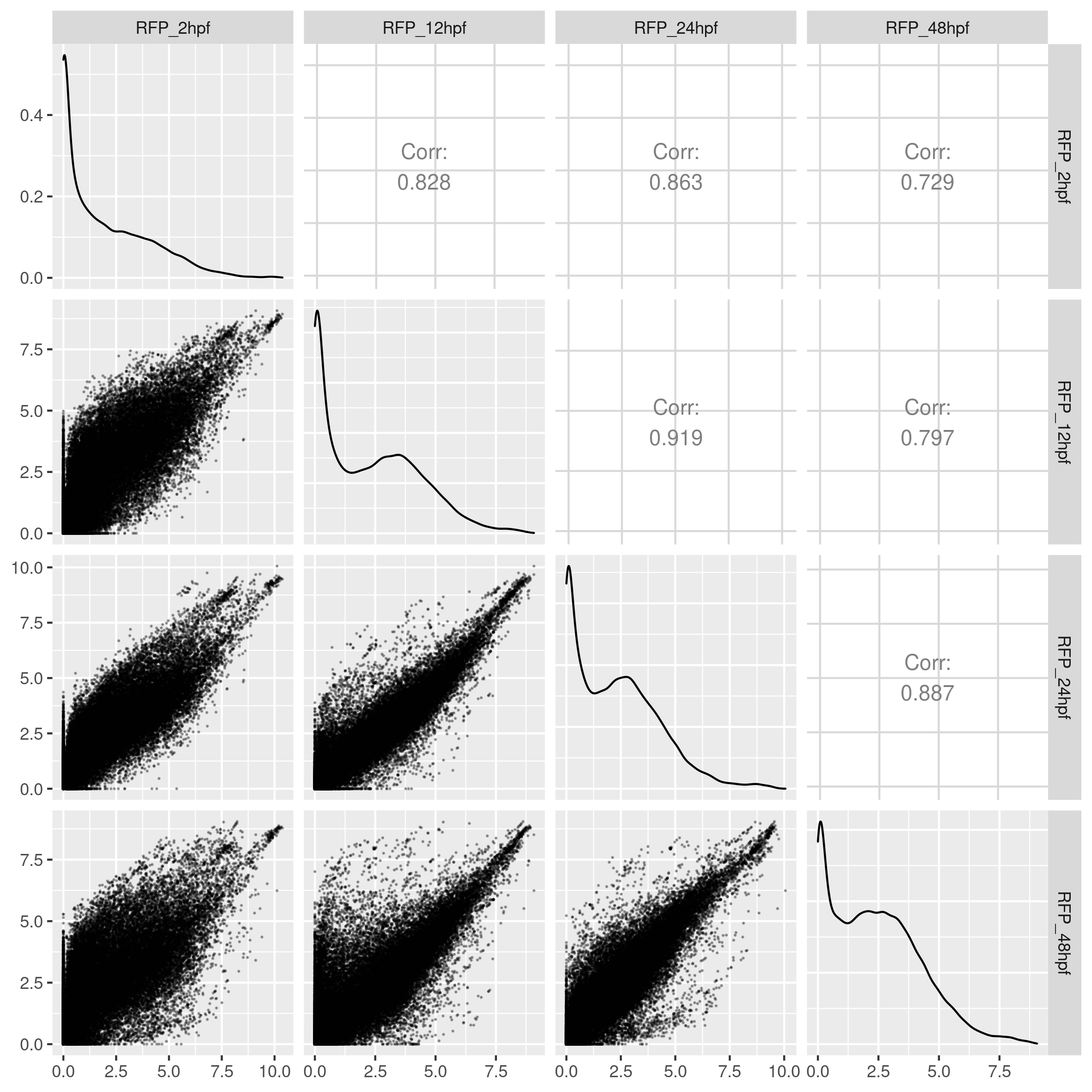
*

[**Figure S5**](#sfigu_cor)***: Correlation plots between all samples.*** *Log2 FPKM correlation of genes between the four stages used of ribo-seq from Bazzini et al 2014* [*[10]*](https://paperpile.com/c/1KmOlc/hAfZU)*. This is part of the default QC.*

*
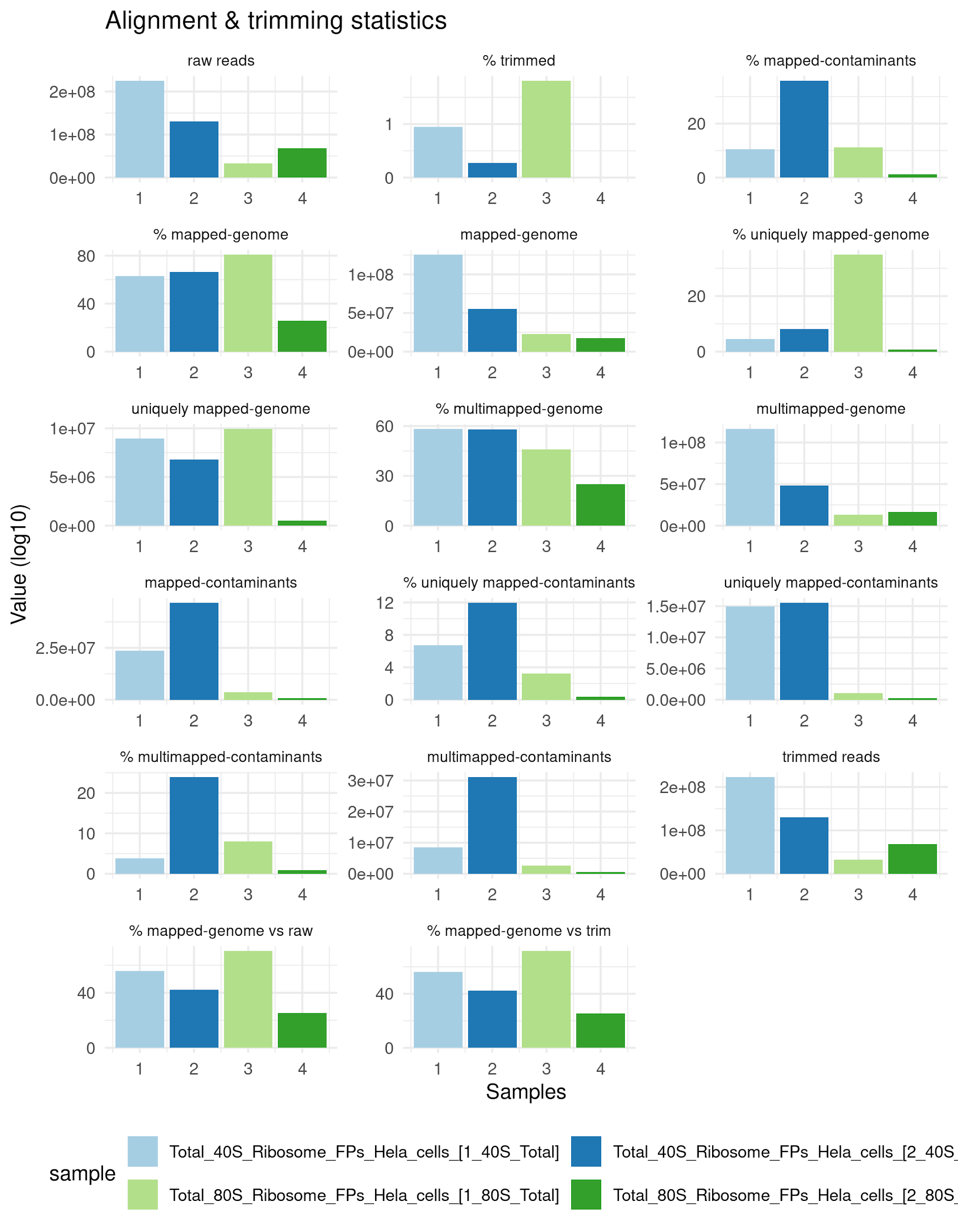
*

[**Figure S6**](#sfigu_align)***: Alignment and trimming statistics.*** *Extracted and visualized output from fastp preprocessing and STAR alignments. Shown are the amount of reads in counts and percentage (%) at the following steps: raw reads from sequencing, trimmed reads (from fastp), contamination depletion (here defined as phix, rRNA, tRNA and non coding RNAs), genome alignment (from STAR). Users will in addition get more detailed FASTQ read statistics from fastp, among others: quality scores, adapter analysis, duplication rates, base content ratios and k-mer counting. Example outputs from fastp can be found here:* [*http://opengene.org/fastp/fastp.html*](http://opengene.org/fastp/fastp.html)*. Data from four samples of TCP-seq from Bohlen et al 2020* [*[19]*](https://paperpile.com/c/1KmOlc/TlsO6)*.*


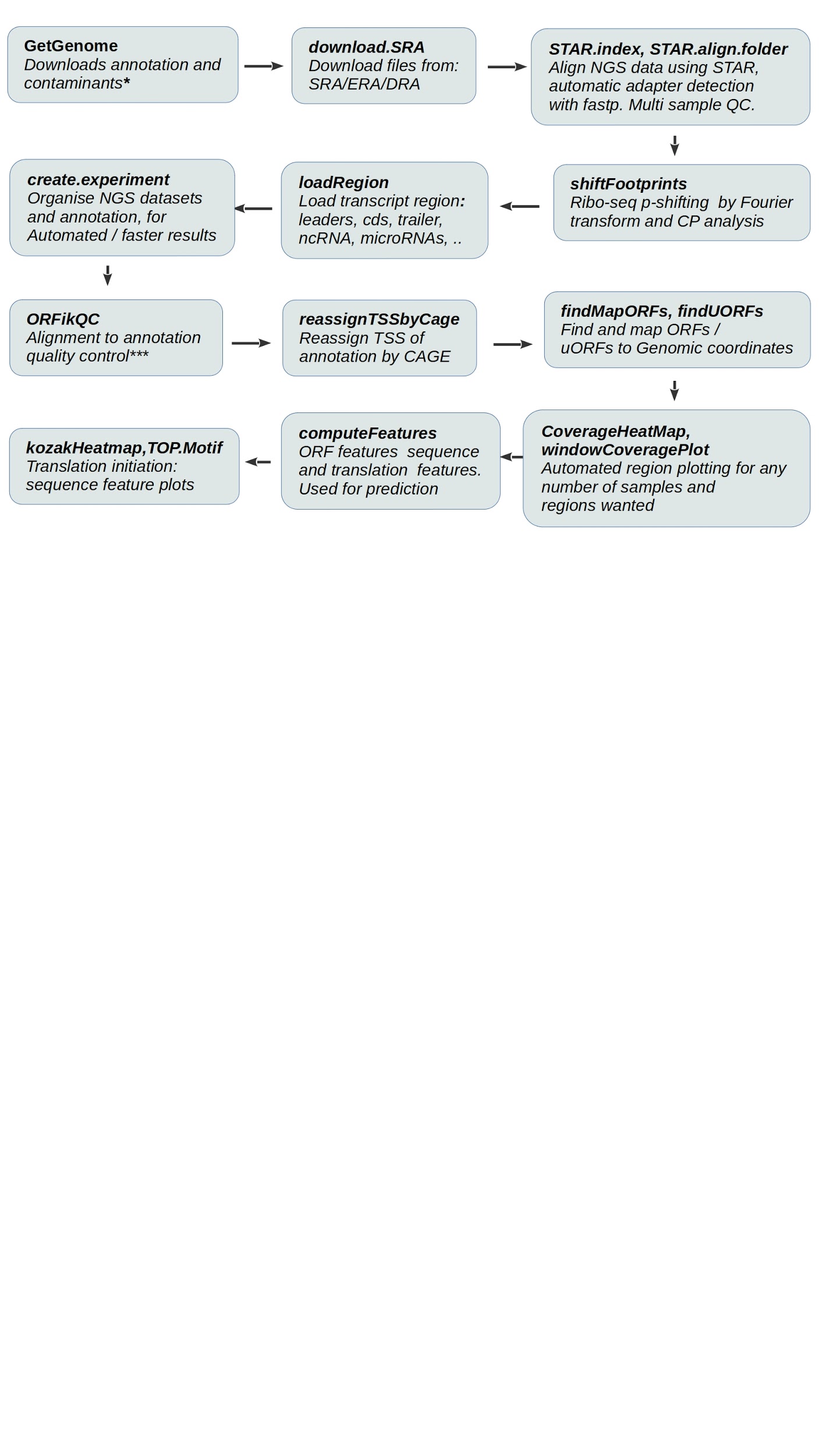


[**Figure S7**](#sfigu_example)***: Example of ORFik workflow.*** *ORFik simplifies and automates all data handling steps, from downloading genome, annotations and experimental data through p-shifting and mapping up to novel ORF detection and classification. * Can download contaminant sequences for depletion in NGS libraries, like Illumina PhiX controls or noncoding RNAs. **Gives direct access to NGS library locations, QC report etc. *** QC report optimized (but not exclusive) for STAR aligner* [*[23]*](https://paperpile.com/c/1KmOlc/DToLL) *and DESeq2* [*[24]*](https://paperpile.com/c/1KmOlc/UMVbW) *.*

*
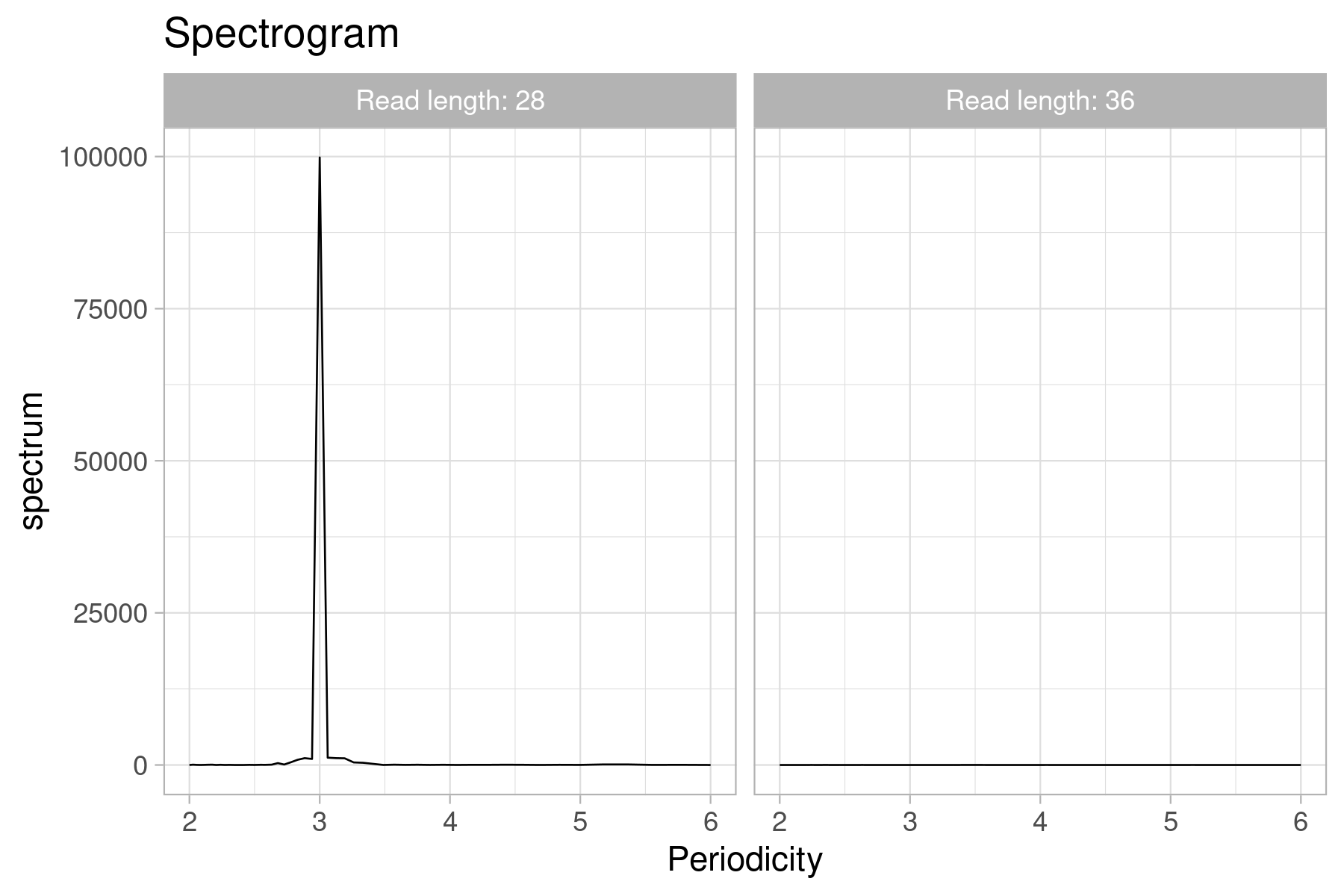
*

[**Figure S8**](#sfigu_spectrum)***: Spectrogram analysis of Ribo-seq read lengths.*** *Spectrograms showing periodicity for two read lengths. The shorter (28nt) shows a 3 nt-periodicity indicative of translation (*[Additional file 1: Figure S10](#sfi_heatmaps)*), while the longer (36nt) does not. Read lengths that do not have a 3 nt read length periodicity will be filtered out for 80S libraries. The peak must be based on at least 1000 reads across the search regions. Data from 2 hpf from Bazzini et al 2014 (*[Additional file 1: Table S9](#sta_data)*)* [*[10]*](https://paperpile.com/c/1KmOlc/hAfZU)*.*

***
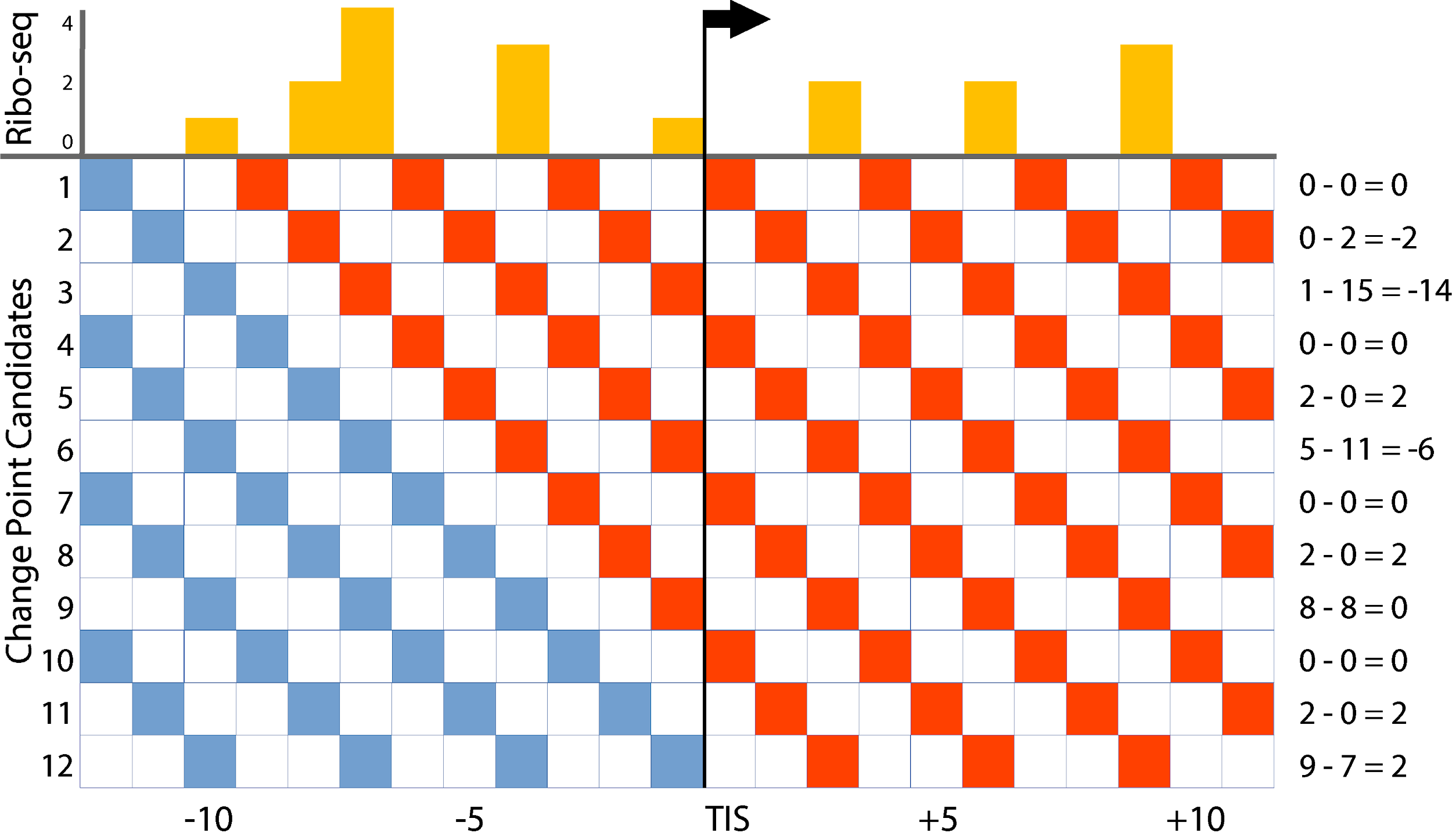
***

[**Figure S9**](#sfigu_pshiftdrawing)***: Graphical explanation of change point analysis during P-site offset-detection in ORFik.***

*Separately for every read length, the 5’ ends of all reads are summed up in a window (+/- 30nt) around the translation initiation site (TIS). From this distribution (yellow) every position in the window (x-axis) is evaluated as a potential change point (y-axis). For each position the sum of the reads in the same translational frame (every 3rd nucleotide) in a window upstream (blue squares) is compared to the sum of reads at the position and downstream (red squares). These sums are given in the right column. The change point with the largest difference, in this case #3 with -14, is selected and the shift is calculated based on the distance between this point and the TIS, in this case 7 nts.*

*
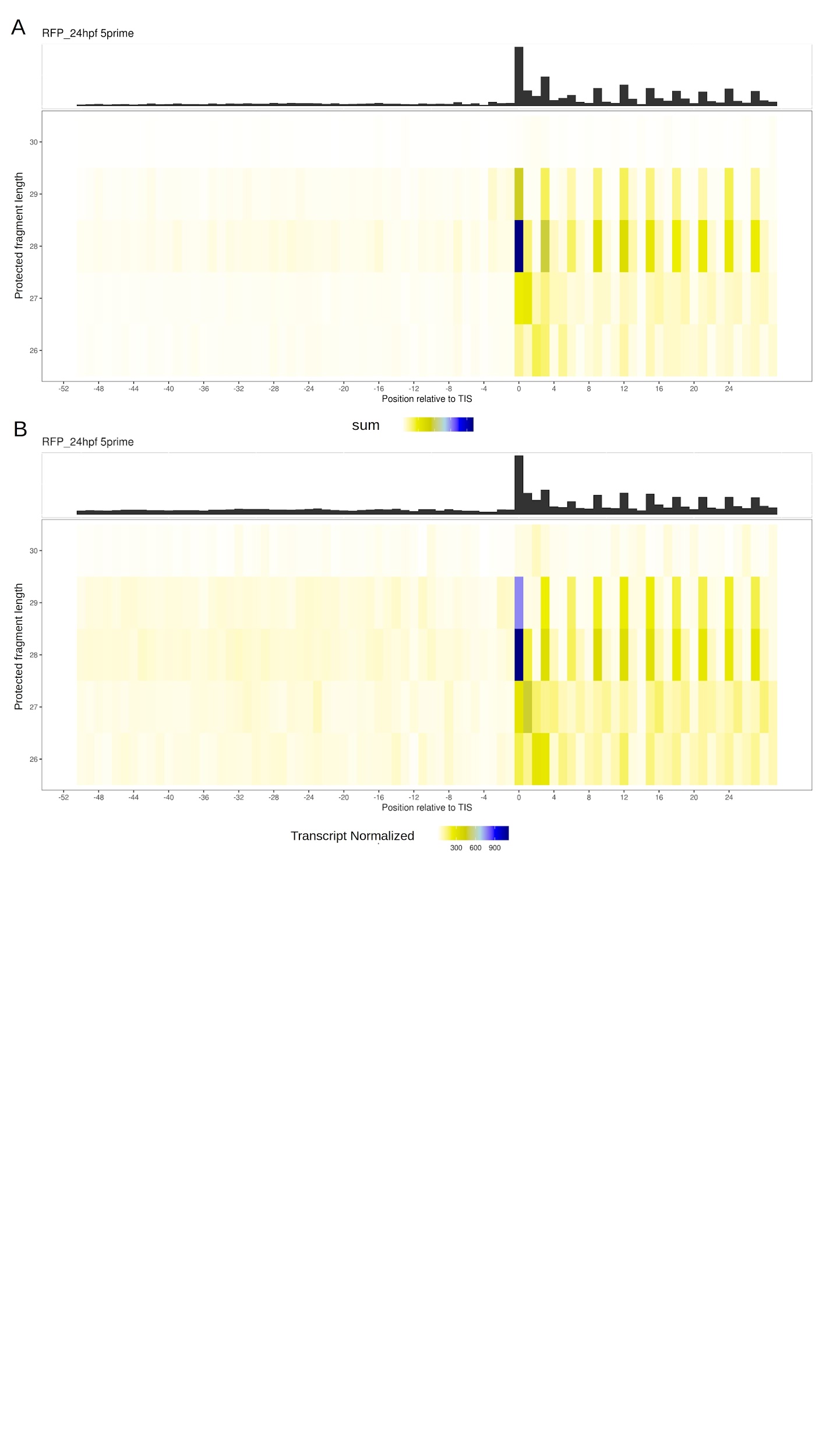
*

[**Figure S10**](#sfigu_heatmaps)***: Meta coverage heatmap around TIS of P-site shifted ribo-seq data using different read count normalization.*** *y-axis:* *Read lengths 26 to 30. x-axis: nucleotide position around TIS (-52 to +29). Colors show A) count of 5’ ends of reads and B) transcript-normalized counts (all counts per gene window sums to 1). Data from Bazzini et al 2014 ribo-seq (*[Additional file 1: Table S9](#sta_data)*)* [*[10]*](https://paperpile.com/c/1KmOlc/hAfZU)*.*

***
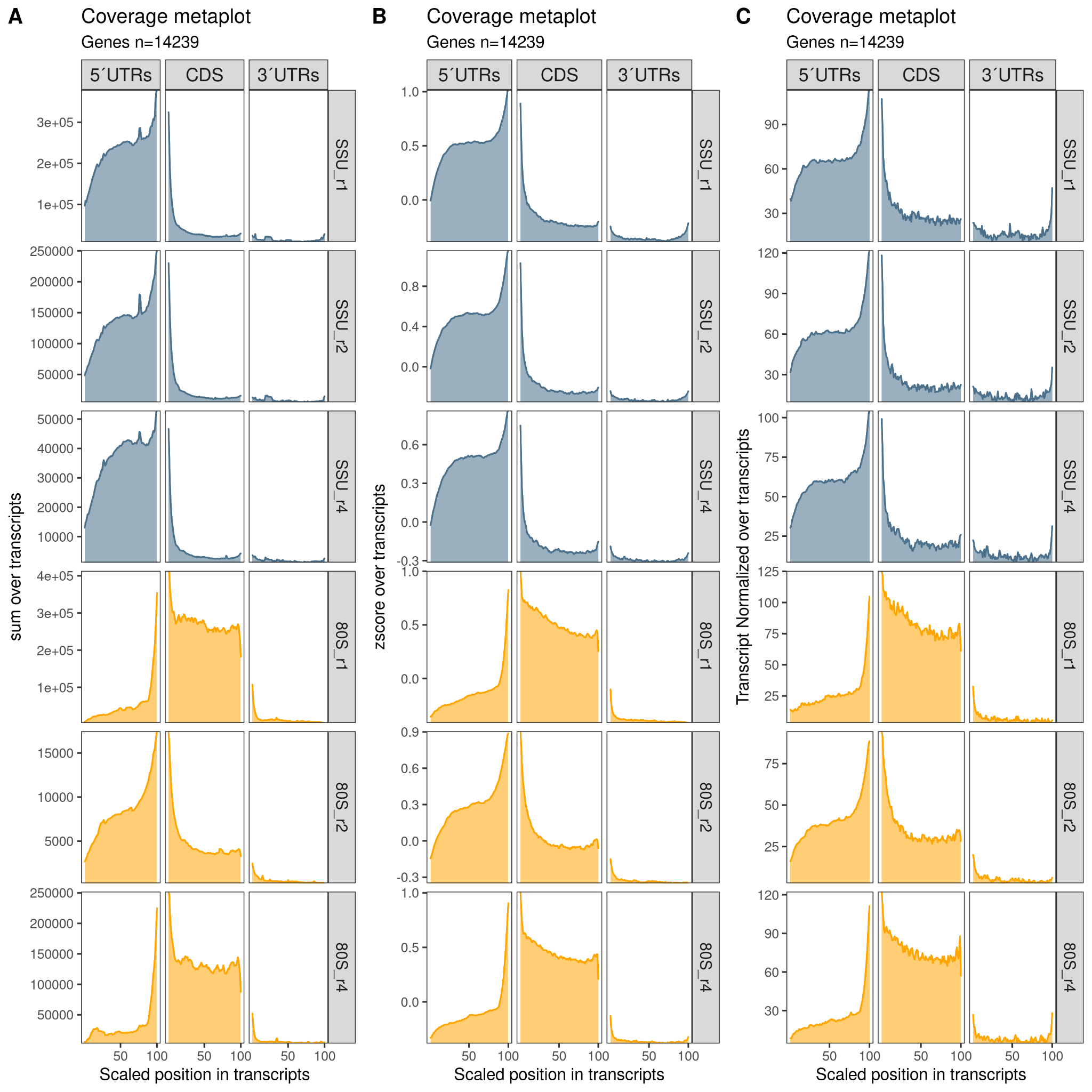
***

[**Figure S11**](#sfigu_meta)***: Meta coverage over mRNAs in non-normalized and normalized counts split by region.*** *Transcripts are split into 5’ UTRs, CDSs and 3’ UTRs. The x-axis shows the relative position normalized to a length of 100 and y-axis shows score value. The scores are* ***A****: Sum (raw counts),* ***B****: z-score (the mean of the z-scores from all transcripts at that position),* ***C****: transcript-normalized (counts from one transcript sums to 1). Colors describe library type; 80S (orange) and SSU (blue). Data from TCP-seq in Bohlen et al 2020 (*[Additional file 1: Table S9](#sta_data)*)* [*[19]*](https://paperpile.com/c/1KmOlc/TlsO6)*.*

***
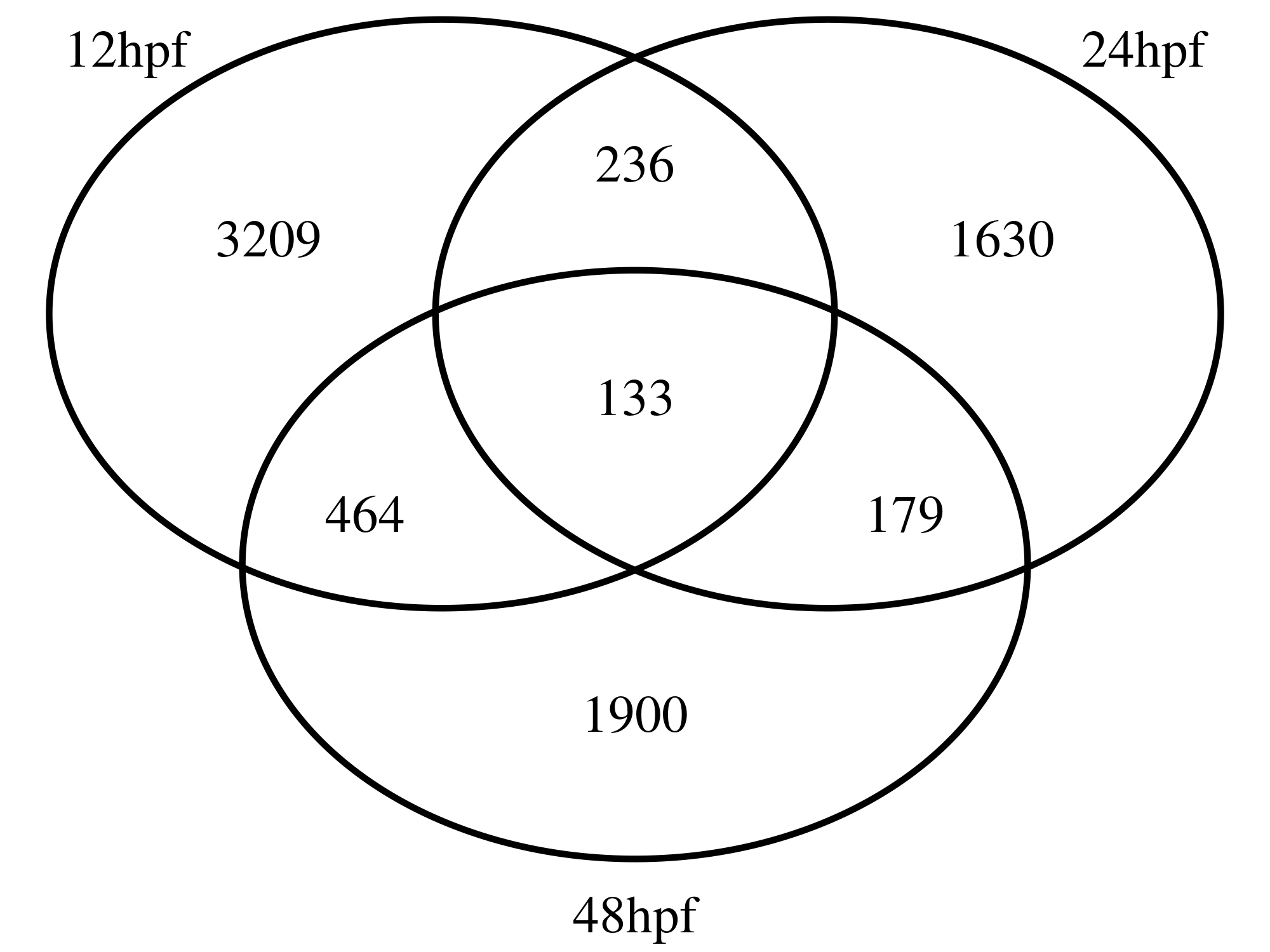
***

[**Figure S12**](#sfigu_venn)***: Predicted uORFs across stages.*** *Venn diagram showing uORFs predicted between stages in the uORF prediction shown in* [Figure 5](#fig_uorf)*.*

**Supplementary Note 1**: Detailed description of uORF prediction pipeline

1. Determine the search-space for uORFs for each CAGE library. The search-space is defined as CAGE derived 5’UTRs + CDS. If CAGE libraries are not included, all CAGE steps are skipped.
2. Find all uORFs in the search-space with default options:

Start codons: "ATG|CTG|TTG|GTG|AAG|AGG|ACG|ATC|ATA|ATT"

Stop codons: “TGA|TAA|TAG”

Minimum length: 6 bases (start and stop codon)

Longest ORF per stop codon: FALSE (all uORFs per stop codon included).

Requirements: Candidate uORF must start in the 5’ UTR and end the base before the CDS stop site. Candidate uORF can’t be in frame relative to the CDS, if the uORF overlaps the CDS.

1. Create unique identifiers for each candidate uORF found. This identifier is defined as transcript id + chromosome + candidate uORF start location + width, separated with an underscore.
2. Find the sequence and NGS features of candidate uORFs using ORFik::computeFeatures function.
3. Store all uORF features in a database (RMysql) for persistence.
4. Create 1 training model and prediction model per stage / cell line. Replicates of the same stage will be grouped together (mean score value over replicates).
5. To create the positive training data, the most highly translated CDS are selected (see below), while for the negative set random untranslated windows in the 3’UTRs are sampled (see below).
   1. For the positive set (CDS) this filtering is applied: (Ribo-seq FPKM > 1 & FPKM > 25th percentile) and (counts > 10 and counts > 25 percentile) and (start codon coverage > 75th percentile) and (periodicity ORFscore > 1). This will train the model conservatively on CDSs that have high coverage around the start codon and a clear periodicity in the Ribo-seq.
   2. For the negative set (3’ UTRs) this filtering is applied: (Ribo-seq FPKM < 75th percentile) or (coverage < 75 percentile) or (start codon coverage < 75th percentile) or (periodicity ORFscore < 0.5). This presents the model with a diverse data set of non-translated sequences that either have little or no coverage, or coverage from overlapping translated ORFs.
6. Assign CDSs that did not pass filters into the negative set.
7. Train the random forest model on the positive and negative sets ([Additional file 1: Table S8](#sta_h20)).
8. Predict uORFs using the model on candidate uORFs with their respective feature data.

*Run time for the uORFome pipeline for the data shown in* [Figure 5](#fig_uorf) *was 14 minutes +/- 2 minutes over 3 runs on CentOS 7 having 196 cores Intel with 2 TB memory. Pipeline used between 1-48 cores depending on the different parts of the pipeline.*

#

# REFERENCES

[1. Trapnell C, Williams BA, Pertea G, Mortazavi A, Kwan G, van Baren MJ, Salzberg SL, Wold BJ, Pachter L: **Transcript assembly and quantification by RNA-Seq reveals unannotated transcripts and isoform switching during cell differentiation**. *Nat. Biotechnol.* 2010, **28**:511–515.](http://paperpile.com/b/1KmOlc/OVcc1)

[2. Ingolia NT, Ghaemmaghami S, Newman JRS, Weissman JS: **Genome-wide analysis in vivo of translation with nucleotide resolution using ribosome profiling**. *Science* 2009, **324**:218–223.](http://paperpile.com/b/1KmOlc/rc1AO)

[3. Ingolia NT, Brar GA, Stern-Ginossar N, Harris MS, Talhouarne GJS, Jackson SE, Wills MR, Weissman JS: **Ribosome profiling reveals pervasive translation outside of annotated protein-coding genes**. *Cell Rep.* 2014, **8**:1365–1379.](http://paperpile.com/b/1KmOlc/xnnIR)

[4. Ji Z, Song R, Regev A, Struhl K: **Many lncRNAs, 5’UTRs, and pseudogenes are translated and some are likely to express functional proteins**. 2015.](http://paperpile.com/b/1KmOlc/tjram)

[5. Calviello L, Mukherjee N, Wyler E, Zauber H, Hirsekorn A, Selbach M, Landthaler M, Obermayer B, Ohler U: **Detecting actively translated open reading frames in ribosome profiling data**. *Nat. Methods* 2015, **13**:165–170.](http://paperpile.com/b/1KmOlc/jxToy)

[6. Zhang S, Hu H, Zhou J, He X, Jiang T, Zeng J: **Analysis of Ribosome Stalling and Translation Elongation Dynamics by Deep Learning**. *Cell Syst* 2017, **5**:212–220.e6.](http://paperpile.com/b/1KmOlc/MOqP3)

[7. Guttman M, Russell P, Ingolia NT, Weissman JS, Lander ES: **Ribosome profiling provides evidence that large noncoding RNAs do not encode proteins**. *Cell* 2013, **154**:240–251.](http://paperpile.com/b/1KmOlc/nnv43)

[8. Chew G-L, Pauli A, Rinn JL, Regev A, Schier AF, Valen E: **Ribosome profiling reveals resemblance between long non-coding RNAs and 5′ leaders of coding RNAs**. *Development* 2013, **140**:2828–2834.](http://paperpile.com/b/1KmOlc/ee7Wz)

[9. Giess A, Jonckheere V, Ndah E, Chyżyńska K, Van Damme P, Valen E: **Ribosome signatures aid bacterial translation initiation site identification**. *BMC Biol.* 2017, **15**:76.](http://paperpile.com/b/1KmOlc/zhJxN)

[10. Bazzini AA, Johnstone TG, Christiano R, Mackowiak SD, Obermayer B, Fleming ES, Vejnar CE, Lee MT, Rajewsky N, Walther TC, Giraldez AJ: **Identification of small ORFs in vertebrates using ribosome footprinting and evolutionary conservation**. *EMBO J.* 2014, **33**:981–993.](http://paperpile.com/b/1KmOlc/hAfZU)

[11. Kumari R, Michel AM, Baranov PV: **PausePred and Rfeet: webtools for inferring ribosome pauses and visualizing footprint density from ribosome profiling data**. *RNA* 2018, **24**:1297.](http://paperpile.com/b/1KmOlc/yiPRW)

[12. Grzegorski SJ, Chiari EF, Robbins A, Kish PE, Kahana A: **Natural Variability of Kozak Sequences Correlates with Function in a Zebrafish Model**. *PLoS One* 2014, **9**:e108475.](http://paperpile.com/b/1KmOlc/kGIOi)

[13. Liu Q, Shvarts T, Sliz P, Gregory RI: **RiboToolkit: an integrated platform for analysis and annotation of ribosome profiling data to decode mRNA translation at codon resolution**. *Nucleic Acids Res.* 2020, **48**:W218–W229.](http://paperpile.com/b/1KmOlc/kPCp)

[14. Alexaki A, Hettiarachchi GK, Athey JC, Katneni UK, Simhadri V, Hamasaki-Katagiri N, Nanavaty P, Lin B, Takeda K, Freedberg D, Monroe D, McGill JR, Peters R, Kames JM, Holcomb DD, Hunt RC, Sauna ZE, Gelinas A, Janjic N, DiCuccio M, Bar H, Komar AA, Kimchi-Sarfaty C: **Effects of codon optimization on coagulation factor IX translation and structure: Implications for protein and gene therapies**. *Sci. Rep.* 2019, **9**:15449.](http://paperpile.com/b/1KmOlc/Bkfb)

[15. Alexaki A, Kames J, Hettiarachchi GK, Athey JC, Katneni UK, Hunt RC, Hamasaki-Katagiri N, Holcomb DD, DiCuccio M, Bar H, Komar AA, Kimchi-Sarfaty C: **Ribosome profiling of HEK293T cells overexpressing codon optimized coagulation factor IX**. *F1000Res.* 2020, **9**:174.](http://paperpile.com/b/1KmOlc/z31b)

[16. **Home - ORFfinder - NCBI** [](http://paperpile.com/b/1KmOlc/FvzAu)<https://www.ncbi.nlm.nih.gov/orffinder/>[].](http://paperpile.com/b/1KmOlc/FvzAu) Accessed 20 May 2020.

[17. Xiao Z, Huang R, Xing X, Chen Y, Deng H, Yang X: **De novo annotation and characterization of the translatome with ribosome profiling data**. *Nucleic Acids Res.* 2018, **46**:e61–e61.](http://paperpile.com/b/1KmOlc/ulkpT)

[18. Nepal C, Hadzhiev Y, Previti C, Haberle V, Li N, Takahashi H, Suzuki AMM, Sheng Y, Abdelhamid RF, Anand S, Gehrig J, Akalin A, Kockx CEM, van der Sloot AAJ, van Ijcken WFJ, Armant O, Rastegar S, Watson C, Strähle U, Stupka E, Carninci P, Lenhard B, Müller F: **Dynamic regulation of the transcription initiation landscape at single nucleotide resolution during vertebrate embryogenesis**. *Genome Res.* 2013, **23**:1938–1950.](http://paperpile.com/b/1KmOlc/oOrWJ)

[19. Bohlen J, Fenzl K, Kramer G, Bukau B, Teleman AA: **Selective 40S Footprinting Reveals Cap-Tethered Ribosome Scanning in Human Cells**. *Mol. Cell* 2020, **79**.](http://paperpile.com/b/1KmOlc/TlsO6)

[20. FANTOM Consortium and the RIKEN PMI and CLST (DGT), Forrest ARR, Kawaji H, et al.: **A promoter-level mammalian expression atlas**. *Nature* 2014, **507**:462–470.](http://paperpile.com/b/1KmOlc/6ciU5)

[21. Chothani S, Adami E, Ouyang JF, Viswanathan S, Hubner N, Cook SA, Schafer S, Rackham OJL: **deltaTE: Detection of Translationally Regulated Genes by Integrative Analysis of Ribo-seq and RNA-seq Data**. *Curr. Protoc. Mol. Biol.* 2019, **129**:e108.](http://paperpile.com/b/1KmOlc/y1XNu)

[22. Li W, Wang W, Uren PJ, Penalva LOF, Smith AD: **Riborex: fast and flexible identification of differential translation from Ribo-seq data**. *Bioinformatics* 2017, **33**:1735–1737.](http://paperpile.com/b/1KmOlc/WaB7W)

[23. Dobin A, Davis CA, Schlesinger F, Drenkow J, Zaleski C, Jha S, Batut P, Chaisson M, Gingeras TR: **STAR: ultrafast universal RNA-seq aligner**. *Bioinformatics* 2013, **29**:15.](http://paperpile.com/b/1KmOlc/DToLL)

[24. Love MI, Huber W, Anders S: **Moderated estimation of fold change and dispersion for RNA-seq data with DESeq2**. *Genome Biol.* 2014, **15**:550.](http://paperpile.com/b/1KmOlc/UMVbW)
